# Supplementary material for: Effect of a freeze-dried coffee solution in a high-fat diet-induced obesity model in rats: Impact on inflammatory response, lipid profile, and gut microbiota
Source: PLoS One. 2022 Jan 26;17(1):e0262270. doi: 10.1371/journal.pone.0262270 (PMC8791513; doi:10.1371/journal.pone.0262270)
Supplement: S1 Table — D: diet; FCS: freeze-dried coffee solution; T: time; SEM: standard error of the mean. (DOCX) [file pone.0262270.s001.docx]

**Table S1.** ***P*-values from the statistical analysis of data of microbial composition and bile acid concentration in the feces of rats fed the control diet without FCS [CT (-)], control diet + FCS [CT (+)], high-fat diet [HF (-)], or high-fat diet + FCS [(HF +)].**

| Item |  |  | *P-*value | | |  | | |
| --- | --- | --- | --- | --- | --- | --- | --- | --- |
|  | D | FCS | T | D × FCS | D × T | FCS × T | D×FCS×T | SEM |
| *Bacteroides spp.* | 0.002 | 0.003 | 0.897 | 0.002 | 0.029 | 0.417 | 0.002 | 0.049 |
| *Lactobacillus spp.* | 0.005 | 0.301 | 0.022 | 0.412 | 0.658 | 0.170 | 0.798 | 0.091 |
| *Enterococcus spp.* | 0.216 | 0.075 | 0.017 | 0.947 | 0.061 | 0.106 | 0.767 | 0.055 |
| *Bifidobacterium spp.* | 0.994 | 0.187 | 0.301 | 0.016 | 0.600 | 0.561 | 0.858 | 0.152 |
| *Escherichia coli* | 0.002 | 0.419 | 0.008 | 0.769 | 0.657 | 0.567 | 0.536 | 0.149 |
| Bile acid | 0.005 | 0.613 | 0.017 | 0.753 | 0.035 | 0.163 | 0.732 | 17.775 |

D: diet; FCS: freeze-dried coffee solution; T: time; SEM: standard error of the mean.
