# Supplementary figures and images for: Effect of a freeze-dried coffee solution in a high-fat diet-induced obesity model in rats: Impact on inflammatory response, lipid profile, and gut microbiota
Source: PLoS One. 2022 Jan 26;17(1):e0262270. doi: 10.1371/journal.pone.0262270 (PMC8791513; doi:10.1371/journal.pone.0262270)

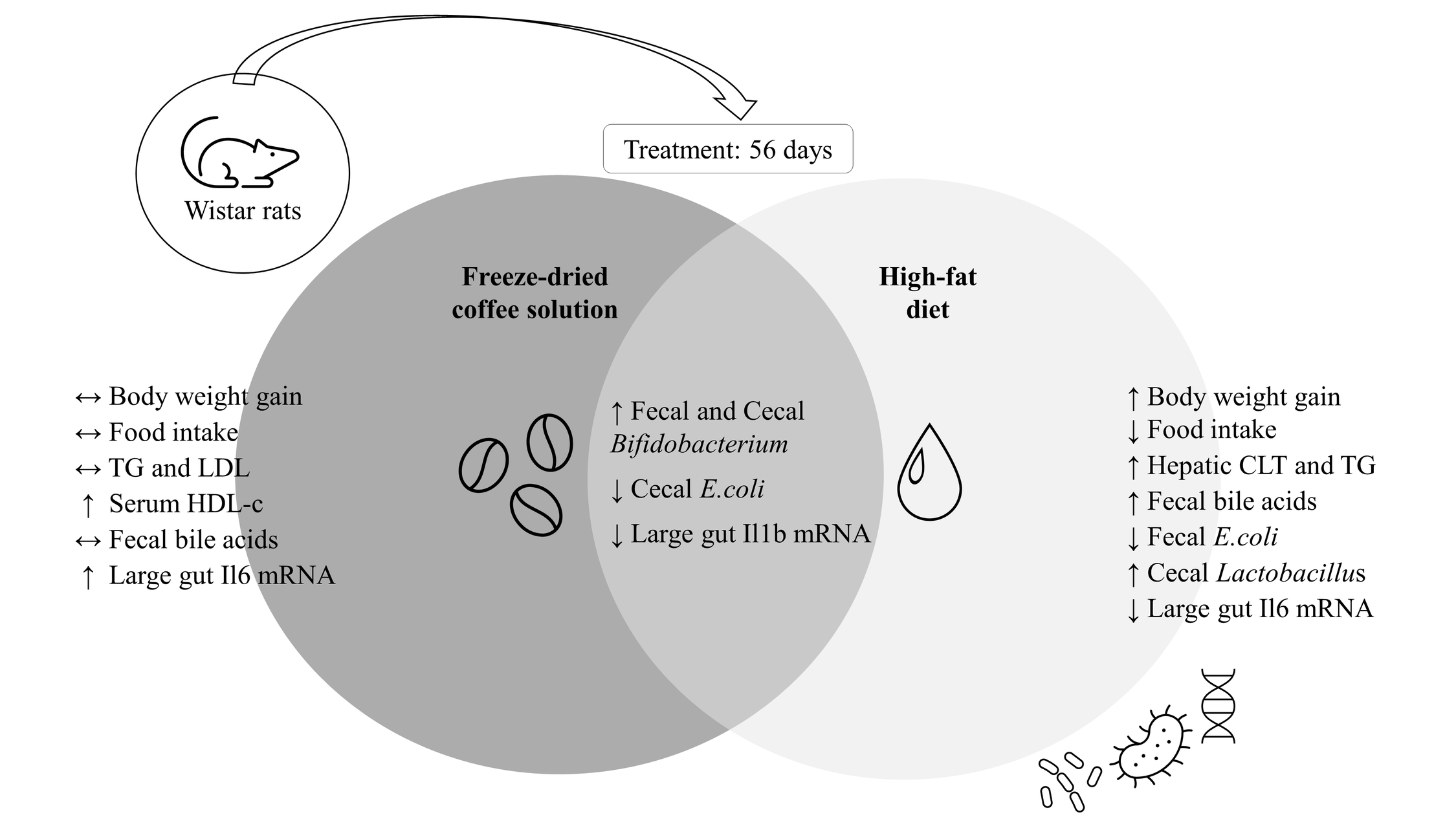

Supplement: S1 Graphical abstract — (TIF) [file pone.0262270.s002.tif]
