# Supplementary material for: Effect of a freeze-dried coffee solution in a high-fat diet-induced obesity model in rats: Impact on inflammatory response, lipid profile, and gut microbiota
Source: PLoS One. 2022 Jan 26;17(1):e0262270. doi: 10.1371/journal.pone.0262270 (PMC8791513; doi:10.1371/journal.pone.0262270)

PLOS ONE

Effect of freeze-dried coffee solution in a high-fat diet-induced obesity model in rats: biochemical and inflammatory impacts and effects on gut microbiota

--Manuscript Draft--

**Manuscript Number:**	PONE-D-21-14462
**Article Type:**	Research Article
**Full Title:**	Effect of freeze-dried coffee solution in a high-fat diet-induced obesity model in rats: biochemical and inflammatory impacts and effects on gut microbiota
**Short Title:**	Freeze-dried coffee solution in rats with high-fat diet: biochemical, inflammatory and gut microbiota impacts
**Corresponding Author:**	Marilia Hermes Cavalcanti<br>Universidade de Brasilia<br>Brasília, DF BRAZIL
**Keywords:**	Coffee; obesity; Gut microbiota; Lipid profile; Bile acids; Intestinal inflammation.
**Abstract:**	Gut microbiota has been linked to the etiology of the obesity process. Foods that are rich in polyphenols seem to positively affect gut microbiota, and consequently prevent obesity and its comorbidities. This study investigates the effect of freeze-dried coffee solution (FCS) consumption on physiological parameters, lipid profile, and microbiota during obesity induction in rats by a high-fat diet. Animals divided into the following 56-day treatment groups: control (CT-); control + FCS (CT+); high-fat diet (HF-); and high-fat diet + FCS (HF+). The FCS combined with a high-fat diet increased fecal and cecal Bifidobacterium populations, decreased cecal E. coli population and intestinal Il1b mRNA levels. Independently of the diet type, the FCS increased serum HDL-c and did not affect body weight, food intake, LDL, triglycerides, fecal bile acids, and intestinal Il6 mRNA levels. The high-fat diet increased weight gain, hepatic cholesterol and triglycerides, fecal bile acids, fecal and cecal Lactobacillus populations, and it reduced food intake, fecal E coli population, and intestinal Il6 mRNA levels. The results suggest that consumption of FCS has a positive effect on health in rats fed with a high-fat diet by increasing Bifidobacterium populations and HDL-C reverse cholesterol transport to tissues, and reducing Il1b mRNA levels. However, the FCS administered in a dose of 0.39g/100g diet over a period of 8 weeks was not effective in controlling food intake and consequently preventing weight gain in the rats during obesity induction by the consumption of a high-fat diet.Gut microbiota has been linked to the etiology of the obesity process. Foods that are rich in polyphenols seem to positively affect gut microbiota, and consequently prevent obesity and its comorbidities. This study investigates the effect of freeze-dried coffee solution (FCS) consumption on physiological parameters, lipid profile, and microbiota during obesity induction in rats by a high-fat diet. Animals divided into the following 56-day treatment groups: control (CT-); control + FCS (CT+); high-fat diet (HF-); and high-fat diet + FCS (HF+). The FCS combined with a high-fat diet increased fecal and cecal Bifidobacterium populations, decreased cecal E. coli population and intestinal Il1b mRNA levels. Independently of the diet type, the FCS increased serum HDL-c and did not affect body weight, food intake, LDL, triglycerides, fecal bile acids, and intestinal Il6 mRNA levels. The high-fat diet increased weight gain, hepatic cholesterol and triglycerides, fecal bile acids, fecal and cecal Lactobacillus populations, and it reduced food intake, fecal E coli population, and intestinal Il6 mRNA levels. The results suggest that consumption of FCS has a positive effect on health in rats fed with a high-fat diet by increasing Bifidobacterium populations and HDL-C reverse cholesterol transport to tissues, and reducing Il1b mRNA levels. However, the FCS administered in a dose of 0.39g/100g diet over a period of 8 weeks was not effective in controlling food intake and consequently preventing weight gain in the rats during obesity induction by the consumption of a high-fat diet.
**Order of Authors:**	Marilia Hermes Cavalcanti
	João Paulo Santos Roseira
	Eliana dos Santos Leandro

Powered by Editorial Manager® and ProduXion Manager® from Aries Systems Corporation

	Sandra Fernandes Arruda
**Additional Information:**	
**Question**	**Response**
**Financial Disclosure**<br><br>Enter a financial disclosure statement that describes the sources of funding for the work included in this submission. Review the submission guidelines for detailed requirements. View published research articles from *PLOS ONE* for specific examples.<br><br>This statement is required for submission and **will appear in the published article** if the submission is accepted. Please make sure it is accurate.<br><br>**Unfunded studies**<br>Enter: *The author(s) received no specific funding for this work.*<br><br>**Funded studies**<br>Enter a statement with the following details:<br>• Initials of the authors who received each award<br>• Grant numbers awarded to each author<br>• The full name of each funder<br>• URL of each funder website<br>• Did the sponsors or funders play any role in the study design, data collection and analysis, decision to publish, or preparation of the manuscript?<br>• **NO** - Include this sentence at the end of your statement: *The funders had no role in study design, data collection and analysis, decision to publish, or preparation of the manuscript.*<br>• **YES** - Specify the role(s) played.<br><br>* typeset	The author Marilia Hermes Cavalcanti received financial scholarship for the Coordenação de Aperfeiçoamento de Pessoal de Nível Superior (CAPES) (code 001) and financial aid for the research by Fundação de Apoio a Pesquisa do Distrito Federal (FAP-DF). The funders had no role in study design, data collection and analysis, decision to publish, or preparation of the manuscript.
**Competing Interests**<br><br>Use the instructions below to enter a competing interest statement for this submission. On behalf of all authors, disclose any competing interests that could be perceived to bias this work—acknowledging all financial support and any other relevant financial or non-financial competing interests.	The authors have declared that no competing interests exist.

Powered by Editorial Manager® and ProduXion Manager® from Aries Systems Corporation

This statement is required for submission and **will appear in the published article** if the submission is accepted. Please make sure it is accurate and that any funding sources listed in your Funding Information later in the submission form are also declared in your Financial Disclosure statement.<br><br>View published research articles from *PLOS ONE* for specific examples.<br><br>**NO authors have competing interests**<br><br>Enter: *The authors have declared that no competing interests exist.*<br><br>**Authors with competing interests**<br><br>Enter competing interest details beginning with this statement:<br><br>*I have read the journal's policy and the authors of this manuscript have the following competing interests: [insert competing interests here]*<br><br>* typeset	
**Ethics Statement**<br><br>Enter an ethics statement for this submission. This statement is required if the study involved:<br><br>• Human participants<br>• Human specimens or tissue<br>• Vertebrate animals or cephalopods<br>• Vertebrate embryos or tissues<br>• Field research<br><br>Write "N/A" if the submission does not require an ethics statement.<br><br>General guidance is provided below. Consult the submission guidelines for detailed instructions. **Make sure that all**	The experimental protocol was approved by the Animal Care and Use Committee of the University of Brasília, under protocol n° 25/2018, approved on 05/08/2018, in accordance with the Brazilian National Council for Animal Experimentation Control (CONCEA) and the Guide for the Care and Use of Laboratory Animals. The animals were anesthetized with 3% isoflurane and euthanized by exsanguination via cardiac puncture.

Powered by Editorial Manager® and ProduXion Manager® from Aries Systems Corporation

information entered here is included in the Methods section of the manuscript.<br><br>**Format for specific study types**<br><br>**Human Subject Research (involving human participants and/or tissue)**<br>• Give the name of the institutional review board or ethics committee that approved the study<br>• Include the approval number and/or a statement indicating approval of this research<br>• Indicate the form of consent obtained (written/oral) or the reason that consent was not obtained (e.g. the data were analyzed anonymously)<br><br>**Animal Research (involving vertebrate animals, embryos or tissues)**<br>• Provide the name of the Institutional Animal Care and Use Committee (IACUC) or other relevant ethics board that reviewed the study protocol, and indicate whether they approved this research or granted a formal waiver of ethical approval<br>• Include an approval number if one was obtained<br>• If the study involved *non-human primates*, add *additional details* about animal welfare and steps taken to ameliorate suffering<br>• If anesthesia, euthanasia, or any kind of animal sacrifice is part of the study, include briefly which substances and/or methods were applied<br><br>**Field Research**<br><br>Include the following details if this study involves the collection of plant, animal, or other materials from a natural setting:<br>• Field permit number<br>• Name of the institution or relevant body that granted permission	
**Data Availability**<br><br>Authors are required to make all data underlying the findings described fully available, without restriction, and from the time of publication. PLOS allows rare exceptions to address legal and ethical	No - some restrictions will apply

concerns. See the PLOS Data Policy and FAQ for detailed information.<br><br>A Data Availability Statement describing where the data can be found is required at submission. Your answers to this question constitute the Data Availability Statement and **will be published in the article**, if accepted.<br><br>**Important:** Stating 'data available on request from the author' is not sufficient. If your data are only available upon request, select 'No' for the first question and explain your exceptional situation in the text box.<br><br>Do the authors confirm that all data underlying the findings described in their manuscript are fully available without restriction?	
**Describe where the data may be found in full sentences. If you are copying our sample text, replace any instances of XXX with the appropriate details.**<br><br>• If the data are **held or will be held in a public repository**, include URLs, accession numbers or DOIs. If this information will only be available after acceptance, indicate this by ticking the box below. For example: *All XXX files are available from the XXX database (accession number(s) XXX, XXX.).*<br>• If the data are all contained **within the manuscript and/or Supporting Information files**, enter the following: *All relevant data are within the manuscript and its Supporting Information files.*<br>• If neither of these applies but you are able to provide **details of access elsewhere**, with or without limitations, please do so. For example:<br><br>*Data cannot be shared publicly because of [XXX]. Data are available from the XXX Institutional Data Access / Ethics Committee (contact via XXX) for researchers who meet the criteria for access to confidential data.*	All relevant data are within the manuscript and its Supporting Information files

The data underlying the results presented in the study are available from (include the name of the third party and contact information or URL).<br>• This text is appropriate if the data are owned by a third party and authors do not have permission to share the data.<br><br>* typeset	
Additional data availability information:	

Powered by Editorial Manager® and ProduXion Manager® from Aries Systems Corporation

Manuscript

Click here to access/download;Manuscript;PLOS ONE Manuscript MHC 042021 .doc

Effect of freeze-dried coffee solution in a high-fat diet-induced obesity model in rats: biochemical and inflammatory impacts and effects on gut microbiota

Marilia Hermes Cavalcanti[1¶*,#a], João Paulo Santos Roseira[2¶,#b], Eliana dos Santos Leandro[1¶,#a], Sandra Fernandes Arruda[1¶,#a]

[1] Postgraduate Program in Human Nutrition, Faculty of Health Sciences, Campus Universitário Darcy Ribeiro, Universidade de Brasília, Brasília, Distrito Federal, Brazil

[2] Department of Animal Science, Universidade Federal de Viçosa, Viçosa, Minas Gerais, Brazil

[#a] Current Address: Postgraduate Program in Human Nutrition, Faculty of Health Sciences, Campus Universitário Darcy Ribeiro, Universidade de Brasília, Brasília, Distrito Federal, Brazil

[#b] Current Address: Department of Animal Science, Universidade Federal de Viçosa, Viçosa, Minas Gerais, Brazil

*Corresponding author

E-mail: mariliaunb@outlook.com (MHC)

[¶]These authors contributed equally to this work.

Abstract

Gut microbiota has been linked to the etiology of the obesity process. Foods that are rich in polyphenols seem to positively affect gut microbiota, and consequently prevent obesity and its comorbidities. This study investigates the effect of freeze-dried coffee solution (FCS) consumption on physiological parameters, lipid profile, and microbiota during obesity induction in rats by a high-fat diet. Animals divided into the following 56-day treatment groups: control (CT-); control + FCS (CT+); high-fat diet (HF-); and high-fat diet + FCS (HF+). The FCS combined with a high-fat diet increased fecal and cecal *Bifidobacterium* populations, decreased cecal *E. coli* population and intestinal Il1b mRNA levels. Independently of the diet type, the FCS increased serum HDL-c and did not affect body weight, food intake, LDL, triglycerides, fecal bile acids, and intestinal Il6 mRNA levels. The high-fat diet increased weight gain, hepatic cholesterol and triglycerides, fecal bile acids, fecal and cecal *Lactobacillus* populations, and it reduced food intake, fecal *E coli* population, and intestinal Il6 mRNA levels. The results suggest that consumption of FCS has a positive effect on health in rats fed with a high-fat diet by increasing *Bifidobacterium* populations and HDL-C reverse cholesterol transport to tissues, and reducing Il1b mRNA levels. However, the FCS administered in a dose of 0.39g/100g diet over a period of 8 weeks was not effective in controlling food intake and consequently preventing weight gain in the rats during obesity induction by the consumption of a high-fat diet.

Introduction

Obesity is a metabolic disease characterized by excessive fat accumulation and low-grade chronic inflammation, caused by an imbalance between food intake and energy expenditure [1,2]. Obesity is a complex process that involves genetic aspects and environmental factors (diet, lifestyle, etc.). It is considered one of the important risk factors for other metabolic diseases, such as insulin resistance, type 2 diabetes, dyslipidemias, fatty liver disease, atherosclerosis, hypertension, and stroke [2–4]. According to the World Health Organization [5], 39% of adults are overweight and 13% are obese worldwide.

Gut microbiota has been linked to the etiology of the obesity process. It can contribute to the host's insulin resistance, the chronic inflammation process, a higher energy harvest from the diet, and body fat deposition through different mechanisms of interaction with the host [3,5,6]. According to Zhao [7], the link between intestinal microbiota and the development of obesity and other metabolic diseases in hosts may be related to the toxic metabolites derived from the gut microbiota.

Evidence suggests that obese individuals have a larger population of the phylum Firmicutes in the gut compared to the phylum Bacterioidetes [6,8] and a lower diversity of intestinal microbiota populations in relation to specific genera of microorganisms [9,10]. Such an imbalance in intestinal microbiota composition leads to individuals developing obesity [6,8].

Different dietary components affect gut microbiota and may consequently promote or prevent obesity and its comorbidities. The consumption of a high-fat diet, particularly rich in saturated fatty acids, increases the proportion of Firmicutes while decreasing Bacteroidetes; it decreases gut microbiota diversity and modifies the bacterial metabolites released [11–13]. Such changes can influence satiety and fat

deposits and increase the secretion of bile acid and pro-inflammatory cytokines, leading to the subsequent development of systemic inflammation, obesity, insulin resistance, and type 2 diabetes [2,11,14,15].

Contrary to what is observed for excessive fat consumption, polyphenol-rich diets have been shown to positively modulate the gut microbiota composition and prevent obesity induced by a high-fat diet [16]. Coffee is an example of a food that is rich in polyphenols and its health effect on obese individuals has been the subject of research [17–19].

Coffee is a complex mixture of chemical compounds and its composition varies according to the species of the coffee bean (*Coffeea arabica* and *Coffea canephora* var. *Robusta*), roasting process, and fermentation process. Polyphenols (chlorogenic acids, alkaloids polyphenols, caffeine, and trigonelline) and melanoidins are considered to be the components of coffee that are most likely to promote health benefits, as 95% of these compounds reach the large intestine and therefore interact with the intestinal microbiota [18,20].

Studies that evaluate the benefits of coffee in rats fed with a high-fat diet have been carried out using coffee extracts, instant coffee, and purified bioactive components of coffee. To date, there are no studies that have evaluated the effect of incorporating coffee into food with the aim of preventing obesity in rats. Freeze drying is considered to be the best method for preserving the chemical properties of foods. Freeze-dried coffee solutions can be seen as a new product with the potential to be incorporated into different foods. Therefore, the aim of this study was to investigate the effect of a freeze-dried coffee solution when mixed with a high-fat diet on the physiological parameters, lipid profile, and microbiota of rats in a high-fat diet-induced obesity model.

2. Materials and methods

2.1 Preparation of freeze-dried coffee solution

A 10% grounded coffee solution, consisting of a blend of grains from the *Coffea arabica* species (Ponto Aralto, Jundiaí, SP, Brazil), was prepared by filtration using 100% cellulose filter paper (Original, n° 103, *Melitta*®, São Paulo, SP, Brazil) and water at 90°C. The coffee solution was freeze-dried using an industrial freeze dryer (Beta 2-8 LSC PLUS Martin Christ, Nova Analítica Ltda, São Paulo, SP, Brazil) and stored at -80ºC until the formulation of the diets. The freeze-dried coffee solution (FCS) was added to the diets at a proportion of 3.9g/kg of diet. The amount of FCS added to the rats' diet is equivalent to 163 ml/day or 1.5 cups/day of coffee, the usual mean coffee intake of the Brazilian population [21]. A previously characterization of the commercial brand *Coffea arabica* species (Ponto Aralto, Jundiaí, SP, Brazil) found 19.93, 4.22, 5.14 and 9.63 mg of caffeine fractions (3-, 4-, 5- caffeoylquinic acid)/g and 18.99 mg of total caffeoylquinic acids/g [22]

2.2. Ethics Statement

The experimental protocol was approved by the Animal Care and Use Committee of the University of Brasília, under protocol n° 25/2018, approved on 05/08/2018, in accordance with the Brazilian National Council for Animal Experimentation Control (CONCEA) and the Guide for the Care and Use of Laboratory Animals [23].

2.3 Animals

Twenty-eight male Wistar rats (Institute of Biomedical Sciences, University of São Paulo, SP, Brazil) aged 21 days old and with an average body weight of 67.37 ± 6.04g were housed individually in stainless steel cages in a room with 12/12h light/dark cycles at 22 ± 1°C. The diet was provided from 12pm to 8am, with free access to water.

The animals were kept for seven days on the AIN-93G control diet [24] for acclimatization, and then were assigned to one of the following experimental groups (six rats/group) and treated for a 56-day period with the corresponding diets: control group (**CT-**): AIN-93G diet; high-fat group (**HF-**): AIN-93G diet with 58% of fat; coffee group (**CF+**): AIN-93G diet with 3.9g of FCS/kg of diet; and high-fat + coffee group (**HF+**): AIN-93G diet with 58% of fat (51.9% of lard and 6.1% of soy oil) and 3.9 g of FCS/kg of diet. The fat percentage was determined according to the Research Diets, Inc. Diet-Induced Obesity Model (D12492, Research Diets, Inc., New Brunswick, NJ, USA).

During the experimental period, the feces of each animal were collected daily, pooled in the same tube for a week, and stored at -80ºC. This provided eight-weeks fecal samples from each animal by the end of the experimental period. These were analyzed in the first and eighth weeks of treatment. Body weight was recorded weekly and food intake daily.

At the end of the treatment period, after a 7h fasting period the animals were anesthetized with 3% isoflurane (BioChimico, Rio de Janeiro, RJ, Brazil) and euthanized by exsanguination via cardiac puncture. The large intestine (ileocecal valve to the rectum) and cecum were excised; the remaining fecal content in the large intestine and the cecum content were collected in sterile tubes and stored at -80°C. Subsequently,

the large intestine was washed in 0.9% saline solution at 4°C, immediately frozen in liquid nitrogen, and stored at -80°C for further analysis.

2.4 Lipid profile

Total cholesterol (CLT), HDL cholesterol, LDL cholesterol, and triglyceride (TG) concentrations were measured in serum and liver using commercial enzymatic/colorimetric kits (BioClin, Belo Horizonte, MG, Brazil) according to the manufacturer's assay protocol. Total lipid extraction from the liver was performed according to Vieira *et al*. [25]. Briefly, 50mg of liver was homogenized in 1ml of isopropanol and centrifuged at 2000 x *g*/10min at 4°C. The supernatant was removed and stored at -80°C until the analysis.

2.5 Bile acid concentration in feces

An aliquot of feces from the first and eighth weeks of treatment was freeze dried at -45°C for 48h. The freeze-dried samples were macerated in a porcelain mortar using liquid nitrogen. Total bile acids were extracted in ethanol according to Kanamoto *et al*. [26] and Tamura *et al*. [27] and the concentration of total bile acids was measured according to a fluorometric method using a commercial assay kit (Sigma-Aldrich, San Luis, MO, USA), following the manufacturer's instructions.

2.6 Determination of fecal microbiota composition

2.6.1 Extraction of DNA from fecal samples and cecum content

The DNA from the feces samples was extracted using a QIAamp PowerFecal DNA kit (Qiagen, Hilden, RP, Germany) according to the manufacturer's assay

protocol, with the following modifications: 150mg of feces were used and the samples were homogenized using a cell/tissue disruptor (L-beader 6, Cotia, São Paulo, SP, Brazil) following a schedule of 2 cycles of 2500rpm/15s for cecum contents and 3 cycles of 2500rpm/15s for feces. The samples were placed on ice for 30s between each cycle.

The DNA samples were quantified by determining the absorbance at 260nm using the equation A_{260nm} x 50 x dilution factor while their purity was assessed by determining the absorbance ratios $A_{260}/A_{280\ nm}$ (~ 1.8-2.0) and $A_{260}/_{230\ nm}$ (~ 2.0) [28] and evaluating the electrophoretic DNA profile on agarose gel.

2.6.2 Real-time PCR analysis

The gut microbiota composition of *Bifidobacterium* spp., *Lactobacillus* spp., *Escherichia coli*, *Bacteroides* spp., and *Enterococcus* was evaluated using quantitative real-time polymerase chain reaction (qPCR, StepOnePlus System, Applied Biosystems, Foster City, CA, USA). Every analysis was performed in triplicate using the Fast SYBR Green Master Mix (Applied Biosystems, Foster City, CA, USA), with each well containing 2µl of sample DNA or standard, sense, and antisense oligonucleotides (Table 1) at a concentration of 100nM and water totaling 10µl. The initial DNA denaturation occurred at 95°C (20s), followed by 40 cycles of denaturation at 95°C (3s), annealing of oligonucleotides, and extension at 59-60°C (30s).

Table 1. Primers and reaction conditions for the bacterial genera analyzed by real-time polymerase chain reaction.

**Microorganisms**		**Primers sequence (5'- 3')**	**T (ºC)**	**Reference**
	F	CATGCCGCGTGTATGAAGAA	59°	[29]
E. coli	R	CGGGTAACGTCAATGAGCAAA		

***Enterococus* spp**	F	CCCTTATTGTTAGTTGCCATCATT	60°	[30]
	R	ACTCGTTGTACTTCCCATTGT		
***Bifidobacterium* spp**	F	AGGGTTCGATTCTGGCTCAG	60°	[31]
	R	CATCCGGCATTACCACCC		
***Lactobacillus* spp**	F	TGGATGCCTTGGCACTAGGA	60°	[32]
	R	AAATCTCCGGATCAAAGCTTACTTAT		
***Bacteroides* spp**	F	GAGAGGAAGGTCCCCCAC	60°	[33]
	R	CGCTACTTGGCTGGTTCAG		

F: forward primer; R: reverse primer; T: annealing temperature

Standard curves were constructed for each experiment using five sequential dilutions of bacterial genomic DNA from pure cultures ranging from 200ng to 0.064ng. The results were expressed as log10 of 16S rRNA copy number/g of feces according to the methodology described by Talarico *et al*. [34]. The different strains used were obtained from the America Type Culture Collection (ATCC) (*Escherichia coli* ATCC 25992; *Enterococcus faecalis* ATCC 19433; *Bacteroides* ATCC 25285), a commercial culture (*Bifidobacterium* spp. BL 04), and from the Tropical Cultures Collection (*Lactobacillus plantarum* UnB SBR64.1 MK5114407).

2.7 Determination of the mRNA levels of the pro-inflammatory genes associated with inflammation

2.7.1 RNA extraction and cDNA synthesis

Total RNA extraction from the large intestine was carried out using TRIzol reagent™ (Invitrogen Inc., Burlington, ON, Canada) according to the manufacturer's assay protocol. Total sample RNA was quantified by measuring the absorbance at 260nm (A_{260nm} x 40 x dilution factor) and its purity was evaluated by the absorbance

ratios A_{260nm}/A_{280nm} and A_{260nm}/A_{230nm} [28]. The integrity of the RNA bands was also verified by their electrophoretic profile on agarose gel.

The complementary DNA (cDNA) synthesis was performed using the High-Capacity cDNA Reverse Transcription Kit with RNase Inhibitor (Applied Biosystems, Foster City, CA, USA).

2.7.2 Quantification of transcript levels of pro-inflammatory genes Il1b, Il6, and Tnfa

The transcript levels of interleukin 1 beta (Il1b), interleukin 6 (Il6), and tumor necrosis factor alpha (Tnfa) in the large intestine were determined by real-time polymerase chain reaction (RT-qPCR). The reaction was performed using 2.0µl of cDNA (final concentration of 20ng), 5.0µl of Fast SYBR Green Master Mix (Applied Biosystems, Foster City, CA), and 0.2µmol/l (final concentration) of each primer (Table 2), in a final volume of 10µl. The qPCR reactions were performed at 95°C (20s) followed by 40 cycles at 95ºC (3s) and 60°C (30s). The primers used are shown in Table 2. All samples were assayed in triplicate, normalized to β-actin as a housekeeping gene, and the amplification specificity of each amplicon was analyzed using the dissociation curve. The relative quantification of each target gene mRNA level was determined using the $2^{-\Delta\Delta CT}$ method [35].

Table 2. Sequences of primers used for RT-qPCR assay of Il1b, Il6, and Tnfa

Gene		Primers sequence (5'- 3')	Reference
**Interleukin 1 beta (Il1b)**	F<br>R	CACCTCTCAAGCAGAGCACAG<br>GGGTTCCATGGTGAAGTCAAC	[36]
**Interleukin 6 (Il6)**	F<br>R	GCCAGAGTCATTCAGAGCAATA<br>GTTGGATGGTCTTGGTCCTTAG	[37]
**Tumor necrosis**	F	AAATGGGCTCCCTCTCATCAGTTC	[36]

**factor alpha (Tnfa)**	R	GTCGTAGCAAACCACCAAGCAGA	
**β Actin**	F<br>R	GTCGTACCACTGGCATTGTG<br>CTCTCAGCTGTGGTGGTGAA	[38]

F: forward primer; R: reverse primer.

2.8 Statistical analysis

The data obtained from microbial populations and bile acids were analyzed according to a completely randomized 2 × 2 × 2 factorial design, considering two diets (CT and HF) with (+) or without (-) the addition of coffee and two treatment periods (1st and 8th week).

The variables from the other experiments were analyzed according to a completely randomized 2 × 2 factorial design (diets and coffee). Homogeneity of the variances between treatments was assumed and after analysis of variance the significant interactions between the factors were unfolded and compared using the F test and Tukey test. The box plot method was used to remove the outlier and the animals' initial weight was used as a covariate. 0.05 was adopted as a critical probability level for type I errors, using PROC MIXED from SAS version 9.4 (SAS Institute Inc., Cary, NC, USA).

3. Results

3.1 Effect of freeze-dried coffee solution consumption on food intake and body weight

The effect of FCS on food intake and body weight gain was measured in the rats treated with the control or and high-fat diet (Table 3). The addition of FCS to the control (CT+) or high-fat diet (HF+) did not significantly affect ($P > 0.05$) food intake or

body weight gain. However, the rats fed a high-fat diet (HF-) showed lower (P <0.05) total and daily food intake and higher weight gain compared to the control rats (CT-).

Table 3. Food intake, final body weight, and average daily weight gain of rats treated with control or high-fat diet with or without freeze-dried coffee solution.

Diet	FCS		Mean ± SE	*P*-value		
	(-)	(+)		Diet	FCS	Diet × FCS
	Total Food Intake (g/56 days)			0.001	0.989	0.218
CT	1,020.51 ± 32.95	1,063.63 ± 33.00	1,042.07 ± 23.22[a]			
HF	893.98 ± 35.46	851.76 ± 32.83	872.87 ± 24.16[b]			
Mean	957.25 ± 24.20	957.69 ± 23.26				
	Food Intake (g/d)			0.001	0.990	0.219
CT	18.22 ± 0.59	18.99 ± 0.59	18.61 ± 0.41[a]			
HF	15.96 ± 0.63	15.21 ± 0.59	15.59 ± 0.43[b]			
Mean	17.09 ± 0.43	17.10 ± 0.42				
	FBW (g)			0.004	0.648	0.447
CT	386.20 ± 15.06	390.84 ± 15.14	388.52 ± 10.64[b]			
HF	459.93 ± 15.06	441.31 ± 15.03	450.62 ± 10.64[a]			
Mean	423.07 ± 10.67	416.08 ± 10.67				
	ADG (g/d)			0.004	0.648	0.447
CT	4.80 ± 0.27	4.88 ± 0.27	4.84 ± 0.19[b]			
HF	6.12 ± 0.27	5.78 ± 0.27	5.95 ± 0.19[a]			
Mean	5.46 ± 0.19	5.33 ± 0.19				

Values are means ± S.E., n = 7/group. Means in a column without a common letter differ (*P* <0.05), according to the F test. CT (-): rats treated with control diet AIN-93G; CT (+): rats treated with control diet AIN-93G + FCS; HF (-) rats treated with high-fat diet; HF (+) rats treated with high-fat diet + FCS; FBW = final body weight; ADG = average daily weight gain.

3.2 Hepatic and serum lipid concentration

The hepatic and serum lipid profiles were determined in the rats treated with the control or high-fat diet, with or without the addition of FCS (Table 4). The FCS intake caused a significant (P <0.05) increase in serum HDL concentration, independently of

the diet type. The high-fat diet promoted an increase of total cholesterol and triglycerides in the liver compared to the control diet.

Table 4. Serum and hepatic lipid profile of rats treated with control or high-fat diet with or without freeze-dried coffee solution.

Diet	FCS		Mean ± SE	*P*-value		
	(-)	(+)		Diet	FCS	Diet × FCS
	CLT serum (mg/dL)			0.562	0.730	0.627
CT	70.22 ± 6.11	71.06 ± 7.53	70.64 ± 5.25			
HF	67.67 ± 9.67	61.63 ± 7.83	64.65 ± 6.81			
Mean	68.94 ± 5.13	66.34 ± 4.96				
	CLT liver (mg/dL)			0.015	0.101	0.409
CT	8.90 ± 2.44	15.14 ± 2.52	12.02 ± 1.83^{b}			
HF	18.57 ± 2.65	20.78 ± 2.37	19.68 ± 1.83^{a}			
Mean	13.74 ± 1.69	17.96 ± 1.69				
	TG serum (mg/dL)			0.869	0.362	0.965
CT	111.20 ± 16.30	96.71 ± 17.85	103.95 ± 12.57			
HF	108.84 ± 17.21	92.90 ± 15.48	100.87 ± 11.78			
Mean	110.02 ± 10.97	94.8 ± 11.69				
	TG liver (mg/dL)			0.038	0.171	0.377
CT	79.34 ± 19.21	123.83 ± 19.82	101.59 ± 14.43^{b}			
HF	146.69 ± 20.91	157.12 ± 18.69	151.91 ± 14.43^{a}			
Mean	113.03 ± 13.31	140.46 ± 13.31				
	HDL serum (mg/dL)			0.863	0.007	0.730
CT	63.62 ± 7.95	86.88 ± 8.15	75.26 ± 5.94			
HF	59.26 ± 8.89	88.06 ± 7.67	73.66 ± 5.94			
Mean	61.44 ± 5.55^{B}	87.47 ± 5.55^{A}				
	HDL liver (mg/dL)			0.415	0.695	0.940
CT	7.53 ± 0.52	7.77 ± 0.54	7.65 ± 0.39			
HF	8.06 ± 0.56	8.23 ± 0.50	8.14 ± 0.39			
Mean	7.80 ± 0.36	8.00 ± 0.36				
	LDL serum (mg/dL)			0.790	0.669	0.358
CT	21.7 ± 3.58	19.87 ± 3.44	20.79 ± 2.57			
HF	17.33 ± 3.69	22.13 ± 3.40	19.73 ± 2.57			
Mean	19.51 ± 2.39	21.00 ± 2.39				
	LDL liver (mg/dL)			0.538	0.743	0.326
CT	4.55 ± 0.64	5.35 ± 0.61	4.95 ± 0.47			
HF	4.69 ± 0.62	4.29 ± 0.64	4.49 ± 0.47			
Mean	4.62 ± 0.42	4.82 ± 0.42				

Values are means ± S.E., n = 5/group. CT (-): rats treated with control diet AIN-93G; CT (+): rats treated with control diet AIN-93G + FCS; HF (-) rats treated with high-fat

diet; HF (+) rats treated with high fat diet + FCS. Means on the same line without a common capital letter [A, B] differ (*P* < 0.05) and means in the same column without a common lowercase letter [a,b] differ (*P* <0.05), according to the F test. CLT = total cholesterol; TG = triglyceride; HDL = high density lipoprotein cholesterol; LDL = low density lipoprotein cholesterol.

3.3 Bile acid concentration in feces

The concentration of bile acids was determined in the feces of the rats treated with the control and high-fat diets, with or without the addition of FCS, at the first and eighth weeks of treatment (Fig. 1). Independently of the presence or absence of FCS in the diets, the concentration of bile acids in the rats' feces was affected by the type of diet and treatment time (weeks). In the first and eighth weeks of the dietary treatment, the fecal concentration of bile acids was significantly (P <0.05) higher in the rats treated with the high-fat diet compared to the control rats. The rats treated with the high-fat di*et al*so showed a significantly (P <0.05) higher concentration of bile acids in their feces in the eighth week of treatment compared to the first week.

Fig 1. Bile acid concentration in feces. Values are least square means of diet x treatment time interaction of bile acid concentration in feces ± S.E., n = 5/group. CT: rats treated with control diet AIN-93G; HF: rats treated with high fat diet. Means without a common capital letter A, B differ (P <0.05) in relation to the effect of diet type on treatment time and means without a common lowercase letter a,b differ (P <0.05) in relation to the effect of treatment time on diet type, according to the F test.

3.4 Determination of fecal microbiota composition

The absolute quantification of specific groups of bacteria was determined in the feces of the rats treated with the control or high fat diet, with or without the addition of FCS (Fig. 2, 3, 4 and 5). The *P*-values of the statical analysis are presented in S1 Table.

The fecal population of *Bacteroides* spp. was the only one that showed a diet x FCS x time interaction effect (Fig. 2). In the first week of treatment, the population of *Bacteroides* spp. in the feces of the rats treated with the high-fat diet (HF-) was significantly (P <0.05) lower compared to the other groups of rats, while the addition of FCS to the high-fat diet (HF+) normalized the fecal *Bacteroides* spp. concentration (CT- x HF+; P >0.05).

In the eighth week of the dietary treatment, there was no significant effect of FCS on the population of *Bacteroides* spp. in the feces of the rats treated with the control or high-fat diet.

Fig 2. *Bacteroides* spp. population in fecal samples. Values are least square means of the diet x FCS x treatment time interaction of *Bacteroides* spp. population in fecal samples of rats treated with control diet without FCS CT (-), control diet + FCS CT (+), high-fat diet HF (-), or high-fat diet + FCS HF (+). Data represent mean log10 16S rRNA gene copies number/g feces. Values are means ± S.E., n = 5/group. Means without a common lowercase letter differ (P < 0.05), according to the Tukey test.

The fecal population of *Bifidobacterium* spp. presented a diet x FCS interaction effect (Fig. 3). The addition of FCS to the high-fat diet (HF+) significantly increased (P <0.05) the population of *Bifidobacterium* spp. in the rats' feces compared to the high-fat

diet without FCS (HF-). The addition of FCS to the control diet did not affect (P > 0.05) the population of *Bifidobacterium* spp.

Fig 3. *Bifidobacterium* population in fecal samples Values are least square means of the diet x FCS interaction of *Bifidobacterium* population in fecal samples of rats treated with control diet without FCS CT (-), high-fat diet HF (-), control diet + FCS CT (+), or high-fat diet + FCS HF (+). Data represent mean log10 16S rRNA gene copies number/g feces. Values are means ± S.E., n = 5/group. Means without a common capital letter A,B differ (P <0.05) in relation to the effect of diet type on coffee consumption and means without a common lowercase letter a,b differ (P <0.05) in relation to the effect of coffee consumption on diet type, according to the F test.

The high-fat diet significantly affected (P <0.05) the populations of *Lactobacillus* spp. and *E. coli* in the rats' feces, regardless of the presence of FCS or the treatment time (Fig.4). The *Lactobacillus* spp. population was significantly (P <0.05) higher in the feces of the rats treated with the high-fat diet (HF), while the *E. coli* population was significantly (P <0.05) lower compared to those treated with the control diet.

Fig 1. Populations of *Lactobacillus* and *Escherichia coli* in fecal samples of rats treated with control or high-fat diet. Data represent mean log10 16S rRNA gene copies number/g feces. Values are means ± S.E., n = 5/group. Within the same bacteria strain, columns without a common letter differ, (P < 0.05) according to the F test.

The treatment time (weeks) with the experimental diets significantly affected (P <0.05) the populations of *Lactobacillus* spp., *Enterococcus* spp., and *E. coli*, regardless

of the dietary fat content or the addition of FCS to the diet (Fig. 5). The populations of *Lactobacillus* spp., *Enterococcus* spp., and *E.coli* were significantly (P <0.05) higher in the rats' feces in the first week of treatment with the experimental diets compared to in the eighth week.

Fig 2. Populations of *Lactobacillus* spp., *Escherichia coli,* and *Enterococcus* spp. in fecal samples. Rats are treated with control or high-fat diet, with or without FCS, after 1st and 8th weeks of treatment. Data represent mean log10 16S rRNA gene copies number/g feces. Values are means ± S.E., n = 5/group. Within the same bacteria strain, columns without a common letter differ (*P* < 0.05), according to the F test.

3.5 Microbiota composition of the cecum content

The populations of *Enterococcus* spp., *Bifidobacterium* spp., and *E. coli* present in the rats' cecum contents were significantly affected (P <0.05) by the addition of FCS to the diets (Table 5). The *Enterococcus* spp. population in the cecum content significantly increased (P <0.05) with the addition of FCS to the control diet (CT+) compared to the control di*et al*one (CT-). The population of *Bifidobacterium* spp. significantly increased (P <0.05) when FCS was added to the high-fat diet (HF+) in relation to the high-fat di*et al*one (HF-), however no difference was observed in the *Bifidobacterium* spp. population when FCS was added to the control diet. The *E. coli* population of the cecum content showed a significant increase (P <0.05) when coffee was added to the control diet, while a significant reduction (P <0.05) was observed when FCS was combined with the high-fat diet, compared to the CT- and HF- diets, respectively. The *Enterococcus* spp. population was not significantly affected (P > 0.05) by the type of diet in which FCS was incorporated. The high-fat diet (HF-) promoted an

increase in the *Enterococcus* spp. population in the cecum content and did not affect *Bifidobacterium* spp. and *E. coli* compared to the control diet (CT-).

The populations of *Bacteroides* spp. and *Lactobacillus* spp. in the cecum contents were not significantly affected (P >0.05) by FCS consumption, independently of whether the control or high-fat diet was used. However, the high-fat diet promoted a significant increase (P <0.05) in the population of *Lactobacillus* spp. in the rats' cecum content.

Table 5. Bacterial population of cecum content of rats treated with control or high-fat diet with or without freeze-dried coffee solution.

Diet	FCS		Mean ± SE	*P*-value		
	(-)	(+)		Diet	FCS	Diet × FCS
	log10 16S rRNA gene copies number/g feces					
	Bacteroides spp.			0.610	0.486	0.784
CT	4.68 ± 0.10	4.78 ± 0.10	4.73 ± 0.07			
HF	4.64 ± 0.11	4.69 ± 0.10	4.67 ± 0.08			
Mean	4.66 ± 0.07	4.73 ±0.07				
	Lactobacillus spp.			0.001	0.338	0.167
CT	3.51 ± 0.14	3.85 ± 0.12	3.68 ± 0.10^{b}			
HF	4.41 ± 0.16	4.33 ± 0.12	4.37 ± 0.10^{a}			
Mean	3.96 ± 0.09	4.09 ± 0.09				
	Enterococcus spp.			0.037	0.194	0.036
CT	0.27 ± 0.14^{Bb}	0.70 ± 0.11^{Aa}	0.49 ± 0.10			
HF	0.93 ± 0.13^{Aa}	0.81 ± 0.11^{Aa}	0.87 ± 0.09			
Mean	0.61 ± 0.08	0.75 ± 0.07				
	Bifidobacterium spp.			0.370	0.001	0.015
CT	1.77 ± 0.25^{Aa}	2.12 ± 0.26^{Aa}	1.94 ± 0.19			
HF	1.33 ± 0.28^{Ba}	3.09 ± 0.24^{Ab}	2.21 ± 0.19			
Mean	1.55 ± 0.17	2.61 ± 0.17				
	Escherichia coli			0.003	0.263	0.003
CT	1.82 ± 0.20^{Ba}	3.02 ± 0.21^{Aa}	2.42 ± 0.15			
HF	1.73 ± 0.22^{Aa}	0.98 ± 0.19^{Bb}	1.36 ± 0.15			
Mean	1.78 ± 0.13	2.00 ± 0.13				

Values are means ± S.E., n = 5/group. CT (-): rats treated with control diet AIN-93G; CT (+): rats treated with control diet AIN-93G + FCS; HF (-) rats treated with high-fat diet; HF (+) rats treated with high-fat

diet + FCS. Means on the same line without a common capital letter ^{A,B} differ (*P* < 0.05) and means in the same column without a common lowercase letter ^{a,b} differ (*P* <0.05), according to the F test.

3.6 Inflammatory genes in the large intestine

The mRNA levels of Il1b, Il6, and Tnfa were evaluated in the rats treated with the control and high-fat diets, with and without FCS (Table 6). The addition of FCS to the high-fat diet (HF+) decreased (P <0.05) the Il1b mRNA levels in the large intestine compared to the high-fat di*et al*one (HF-), while no difference was observed between the CT- and CT+ groups. Regarding the Il6 mRNA levels in the large intestine, FCS increased these regardless of the diet type, while the high-fat diet promoted a decrease, independently of whether or not FCS was used. The Tnfa mRNA levels in the large intestine were not significantly affected (P> 0.05) by the diet type or presence of FCS.

Table 6. **Quantification of interleukin-1beta (Il1b), interleukin-6 (Il6), and tumor necrosis factor alpha (Tnfa) mRNA levels in the large intestine of rats treated with control or high fat diet with or without freeze-dried coffee solution.**

Diet	Coffee		Mean ± SE	*P*-value		
	(-)	(+)		Diet	Coffee	Diet × Coffee
	mRNA Il1b			0.068	0.049	0.006
CT	1.00 ± 0.09^{Aa}	1.19 ± 0.09^{Aa}	1.10 ± 0.06			
HF	1.21 ± 0.10^{Aa}	0.63 ± 0.10^{Bb}	0.91 ± 0.07			
Mean	1.11 ± 0.06	0.91 ± 0.06				
	mRNA Il6			0.025	0.018	0.825
CT	1.02 ± 0.23	1.62 ± 0.21	1.32 ± 0.16^{a}			
HF	0.53 ± 0.19	1.04 ± 0.21	0.90 ± 0.14^{b}			
Mean	0.78 ± 0.15^{B}	1.33 ± 0.15^{A}				
	Tnfa			0.849	0.580	0.287
CT	0.97 ± 0.22	0.87 ± 0.22	0.92 ± 0.15			
HF	0.72 ± 0.18	1.05 ± 0.18	0.88 ± 0.13			
Mean	0.85 ± 0.14	0.96 ± 0.14				

Values are means ± S.E., n = 6/group. CT (-): rats treated with control diet AIN-93G; CT (+): rats treated with control diet AIN-93G + FCS; HF (-) rats treated with high-fat diet; HF (+) rats treated with high-fat

diet + FCS. Means on the same line without a common capital letter[A,B] differ (P < 0.05) and means in the same column without a common lowercase letter[a,b] differ (P <0.05), according to the F test.

4. Discussion

This study evaluated the effect of FCS when mixed with the diet on the physiological parameters, lipid profile, bile acids, and microbiota of rats in a high-fat diet-induced obesity model.

Several studies have shown the effectiveness of coffee extract in reducing body weight gain in rats treated with a high-fat diet [39–44]. Polyphenols, which are present in a high concentration in coffee, are able to inhibit digestive enzymes [45] and consequently inhibit macronutrient absorption and reduce body weight gain [16,20]. Contrary to what has been observed in some studies, in the present study the consumption of FCS for 56 days did not change body weight in the control group or high-fat diet rats. This result suggests that in a treatment model for preventing obesity, when FCS is co-administrated during the development of obesity, a longer treatment time may be necessary to observe the effect of FCS on body weight. A previous study that monitored obesity induction over 83 days by a high-fat diet showed that although the rats on the high-fat diet had a higher body weight than the control rats after day 21 of the treatment, statistical significance was reached only after 83 days [46]. This reinforces the hypothesis that in the present study the rats were in a pre-obesity state. Furthermore, several studies that have observed significant effects of coffee on body weight [39,41,43] have used an obesity treatment model instead of an obesity prevention one as in the present study.

The higher body weight of the rats treated with the high-fat diet despite their lower food intake is associated with the higher energy density of the high-fat diet (5.30

kcal/g) compared to the control (3.95 kcal/g). Even though the dietary intake of rats treated with the high-fat diet was 16% less than the control rats, their energy intake was 13% greater. Similar results have been observed in other studies [47–49]. The authors argue that the decrease in food intake promoted by the high-fat diet may be attributed to a compensatory mechanism that attempts to maintain energy balance homeostasis and consequently control the excessive body weight gain induced by the high caloric density of the diet.

Several other factors, such as coffee bean species (*Coffeea arabica* and *Coffea canephora* var. *Robusta*), roasting process (time x temperature) [50], and the method used to prepare the beverage, influence coffee polyphenol content. Rendon *et al*. [51] showed that filtered coffee beverages have lower diterpene contents than unfiltered ones. According to Cruz *et al*. [52], variations in the biological activity of coffee may be related to such differences in the chemical composition of the beverage.

In the present study, the coffee solution was freeze-dried to enable its incorporation into the high-fat diet. Although this process may promote chemical alterations in the polyphenols, such modifications can be considered minimal, as freeze drying is the most recommended method for preserving polyphenol compounds [53,54]. Another aspect to be considered is the dose of FCS used in the present study (0.39g/100g of diet), which is equivalent to 163 ml/day or 1.5 cups/day of coffee, the usual mean coffee intake of the Brazilian population [21]. When testing two doses of coffee extract (40 and 60% v/v), Maki *et al*. [55] only observed a significant reduction in body weight gain and the amelioration of some biochemical markers in the mice treated with the high dose of 60% coffee extract. Therefore, it is possible that the dose used in the present study was insufficient to reduce the rats' body weight.

Regarding lipide profile, there is no consensus concerning the effect of coffee [43,56–58]. In the present study, the consumption of FCS did not affect most of the lipid profile markers in the serum and liver, since the cholesterol, triglyceride, and LDL levels were similar between the control and high-fat diets, with and without the addition of FCS. Similar to our results, Karabudak *et al.* [59] demonstrated no significant association between Turkish or instant coffee consumption in subjects. The authors suggested that the coffee preparation method and the amount consumed are important aspects in serum lipid response. The cause of differences is not clear but may be associated with the amount of coffee administrated, different routes of coffee administration (gavage or mixed with the diet), and different treatment protocols, as some studies have employed an obesity treatment protocol rather than an obesity prevention one. Ilmiawati [44] *et al.* observed a decrease in serum cholesterol and triglycerides with low doses of a green coffee extract (GCE), however only the high doses of GCE (> 20 mg/kg BWG/d) were able to decrease LDL and increase HDL in serum.

Although FCS did not decrease total cholesterol or the atherogenic LDL lipoprotein, coffee consumption did promote a significant increase in serum HDL lipoproteins. This result shows that coffee has atheroprotective properties, as HDL lipoproteins remove excess cholesterol by reverse cholesterol transport to tissues [60]. This is also in line with the marginal increase in total cholesterol in the liver observed with the coffee treatment (1.31-fold compared to no coffee; $p = 0.100$). Similar to our result, Feyisa *et al.* [56] observed that rats treated with coffee had higher HDL-c concentrations. Some studies, most of which use purified compounds, show that some polyphenols found in coffee (caffeic, ferulic, and phenolic acids), as well as in other

foods, stimulate reverse cholesterol transport by promoting HDL formation and cholesterol efflux [61,62].

Contrary to what was expected, in the rats treated with the high-fat diet no change was observed in serum lipid profiles, and only the hepatic concentration of cholesterol and triglycerides increased. We hypothesize that the obesity induction time was not enough to surpass systemic homeostasis, therefore excess cholesterol was taken up by the liver through LDL receptors mediated by the SCAP-SREBP pathway [63], which caused an increase in hepatic cholesterol, while serum levels remained similar to those of the control rats. The higher bile acid excretion in stools observed in the first week of treatment, which was even greater in the eighth week, in the rats treated with the high-fat diet regardless of FCS consumption, reinforces the hypothesis that systemic homeostasis was maintained until 56 days of treatment.

Bile acids are amphipathic sterols secreted in the duodenum. They are the main constituent of bile and are responsible for emulsifying fat and facilitating digestion and absorption [64]. As expected, the high-fat diet promoted an increase of total bile acids in the rats' feces. Lin *et al*. [11] found lower concentrations of conjugated bile acids and higher levels of unconjugated bile acids in the feces of rats treated with a high-fat diet. Gut microbiota is capable of hydrolyzing conjugated bile acids into secondary bile acids, which are associated with colon cancer [65,66]. Although coffee and its polyphenolic compounds can decrease dietary lipid digestion by reducing the synthesis and action of bile acids [67,68], in the present study the FCS did not affect total bile acid excretion in the feces.

Certain strains of the *Bifidobacterium* genus engage in anti-obesity activity and are therefore found in lower populations in obese individuals. The increase in *Bifidobacterium* spp. in the feces and cecal content promoted by the FCS only in the

rats treated with the high-fat diet suggests that the consumption of FCS during the process of obesity development and progression may have a protective effect, since these rats also presented lower mRNA levels of the pro-inflammatory Il1b in the large intestine. These effects may be associated with the high polyphenol content of coffee (chlorogenic acids, caffeine, cafestol, kahweol), which present anti-inflammatory properties [18,69]. Some studies have shown that polyphenols in foods have prebiotic effects, favoring the *Bifidobacterium* spp. population in the gut [70,71]. Fermentation products from coffee components may inhibit the growth of other microorganisms in the colon, and consequently favor an increase in the *Bifidobacterium* genus in rats treated with coffee combined with a high-fat diet. Nakayama and Oishi [31] showed an increase in *Bifidobacterium* spp. in the feces of rats treated with coffee extract, thus corroborating with our results.

Although the *Lactobacillus* genus is highly associated with host health benefits, some subspecies appear to be positively associated with body weight gain [8]. The high body weight gain observed in the rats treated with the high-fat diet in the present study may be associated with some subspecies of the *Lactobacillus* population generally observed in obese organisms, since a significant increase in *Lactobacillus* spp. was observed in the feces of these rats. Similar to our results, Cowan *et al*. [39] did not observe any effect of coffee administration on the fecal population of *Lactobacillus* spp.

E. coli is generally found in the human gastrointestinal tract, either as commensal, probiotic, or pathogen bacteria [72], comprising less than 0.1% of total bacterial cells in the gut microbiota [73]. The present study observed a reduction in fecal *E. coli* populations with the high-fat diet treatment, while no FCS effect was observed. Corroborating our findings, Million *et al*. [74] suggested that the absence of *E. coli* was an independent predictor of weight gain, as they found *E. coli* in the feces of

patients with weight loss. In vitro studies have shown that phenolic compound extract modulates *E. coli* growth [75–77]. Therefore, in the present study coffee polyphenols or the products of their hydrolization or reduction may have stimulated the growth of *E. coli* in the cecum of the rats fed with the control diet. In line with data from the literature, *Lactobacillus* spp., *E. coli*, and *Enterococcus* spp. populations decreased in the feces with increased treatment time, independent of the dietary treatment employed. Given the wide density and diversity of microorganisms in the gastrointestinal tract, especially in the colon, the release/production of different second metabolites by these microorganisms (organic acids and short chain fatty acids) may explain the reduction of some bacterial strains, since these compounds act as antimicrobial agents [6,78]. Therefore, the longer dietary treatment time may have increased the antimicrobial action of some metabolites on *Lactobacillus*, *E. coli*, and *Enterococcus*, reducing these bacterial populations in the feces.

Lachnospira, *Roseburia*, *Butyrivibrio*, *Ruminoccus*, *Fecalibacterium*, and *Fusobacteria* constitute the main microorganisms in the cecum [79]. However, in the present study, *E. coli*, *Enterococcus* spp., *Lactobacillus* spp., *Bacteroides* spp., and *Bifidobacterium* spp. were identified in the cecum. According to Liu *et al*. [16], *Enterococcus* and *Bifidobacterium* hydrolyze food polyphenols. Therefore, the observed increase in *Bifidobacterium* and *Enterococcus* in the cecum of the rats treated with FCS combined with the control diet and FCS combined with the high-fat diet compared to the control and high-fat diets alone, respectively, may be associated with polyphenols from coffee. In addition to the effect of FCS, it is likely that some fecal strains of *Bifidobacterium* present mechanisms of resistance to bile acids, which results in an increase in its population. In some strains, such as *Bifidobacterium logum* and

Bifidobacterium brevis, mechanisms of resistance to bile acids involving bile efflux systems have already been identified [80–82].

Considering that pro-inflammatory cytokines such as Il6, Il1b, and Tnfa are increased in obese individuals [83] and that consuming four or more cups of coffee/day seems to decrease serum Il6 levels [84], in our study we determined the mRNA levels in the rats' large intestine in order to investigated a possible coffee x fecal microbiota x gut inflammatory response interaction. Despite the high polyphenol content in coffee, the Il6 mRNA levels were increased by coffee consumption regardless of the diet type, while the high-fat diet decreased Il6 mRNA levels in the large intestine. The decrease in Il6 mRNA levels in the large intestine promoted by the high-fat diet in the rats may be related to the inhibition of the *E. coli* population observed in these rats compared to the control. According to Kittana *et al*. [85], a greater population of commensal *E. coli* has been observed in the intestinal tissue of humans during the inflammation process. These authors also demonstrated that some strains of *E. coli* in the intestine are associated with high levels of Il6 secretion.

Despite the accepted idea that foods rich in polyphenols inhibit pro-inflammatory cytokine secretion [86], in the present study coffee promoted an increase in Il6 mRNA levels in the large intestine regardless of the diet type. This contradiction may be attributed to differences in tissue response. Most studies evaluate systemic inflammation by measuring serum levels, while in the present study we evaluated inflammatory response in the large intestine. According to Juge-Aubry *et al*. [87], Il6 may engage in anti-inflammatory activity, since it is able to decrease Tnfa and IFNγ (interferon gamma) over the course of inflammation, in an attempt to control the inflammation and inhibit tissue damage. Caro-Gómez *et al*. [88] observed increased expression levels of hepatic Il6 in rats treated with green coffee extract. The authors

mention that Il6 is important to control obesity-associated inflammation by favoring macrophage polarization towards the M2 phenotype, which acts in the resolution phase of inflammation and in repairing damaged tissues.

Conclusions

The results suggest that the consumption of FCS may promote positive health effect in rats fed with a high-fat diet by increasing the populations of *Bifidobacterium*, improving HDL-c reverse cholesterol transport to tissues, and reducing Il1b mRNA levels. However, FCS administered in a dose equivalent to the usual mean coffee intake of the Brazilian population over an eight-weeks period was not effective in controlling food intake and consequently preventing weight gain in rats during obesity induction by the consumption of a high-fat diet.

References

1. Gregor MF, Hotamisligil GS. Inflammatory Mechanisms in Obesity. Annu Rev Immunol. 2011;29: 415–445. doi:10.1146/annurev-immunol-031210-101322
2. Hotamisligil GS. Inflammation and metabolic disorders. Nature. Nature Publishing Group; 2006. pp. 860–867. doi:10.1038/nature05485
3. Cuevas-Sierra A, Ramos-Lopez O, Riezu-Boj JI, Milagro FI, Martinez JA. Diet, Gut Microbiota, and Obesity: Links with Host Genetics and Epigenetics and Potential Applications. Advances in Nutrition. Oxford University Press; 2019. pp. S17–S30. doi:10.1093/advances/nmy078
4. Mendez-Sanchez N, Cruz-Ramon VC, Ramirez-Perez OL, Hwang JP, Barranco-Fragoso B, Cordova-Gallardo J. New aspects of lipotoxicity in nonalcoholic steatohepatitis. International Journal of Molecular Sciences. MDPI AG; 2018. doi:10.3390/ijms19072034
5. WHO (Word Health Organization). Obesity and overweight. [cited 1 Feb 2021]. Available: https://www.who.int/en/news-room/fact-sheets/detail/obesity-and-overweight
6. Al-Assal K, Martinez AC, Torrinhas RS, Cardinelli C, Waitzberg D. Gut microbiota and obesity. Clinical Nutrition Experimental. Elsevier Ltd; 2018. pp. 60–64. doi:10.1016/j.yclnex.2018.03.001
7. Zhao L. The gut microbiota and obesity: From correlation to causality. Nature Reviews Microbiology. Nat Rev Microbiol; 2013. pp. 639–647.

doi:10.1038/nrmicro3089
8. Cao SY, Zhao CN, Xu XY, Tang GY, Corke H, Gan RY, *et al*. Dietary plants, gut microbiota, and obesity: Effects and mechanisms. Trends in Food Science and Technology. Elsevier Ltd; 2019. pp. 194–204. doi:10.1016/j.tifs.2019.08.004
9. Carlucci C, Petrof EO, Allen-Vercoe E. Fecal Microbiota-based Therapeutics for Recurrent Clostridium difficile Infection, Ulcerative Colitis and Obesity. EBioMedicine. Elsevier B.V.; 2016. pp. 37–45. doi:10.1016/j.ebiom.2016.09.029
10. Le Chatelier E, Nielsen T, Qin J, Prifti E, Hildebrand F, Falony G, *et al*. Richness of human gut microbiome correlates with metabolic markers. Nature. 2013;500: 541–546. doi:10.1038/nature12506
11. Lin H, An Y, Tang H, Wang Y. Alterations of Bile Acids and Gut Microbiota in Obesity Induced by High Fat Diet in Rat Model. J Agric Food Chem. 2019;67: 3624–3632. doi:10.1021/acs.jafc.9b00249
12. Yan X, Wang F, Weng P, Wu Z. The effect of fermented Huyou juice on intestinal microbiota in a high- fat diet- induced obesity mouse model. J Food Biochem. 2020;44: e13480. doi:10.1111/jfbc.13480
13. Zhang M, Yang X-J. Effects of a high fat diet on intestinal microbiota and gastrointestinal diseases. World J Gastroenterol. 2016;22: 8905. doi:10.3748/wjg.v22.i40.8905
14. Cândido FG, Valente FX, Grześkowiak ŁM, Moreira APB, Rocha DMUP, Alfenas R de CG. Impact of dietary fat on gut microbiota and low-grade systemic inflammation: mechanisms and clinical implications on obesity. Int J Food Sci Nutr. 2018;69: 125–143. doi:10.1080/09637486.2017.1343286
15. Kim B, Kim YY, Jung H, Nam H, Suh JG. Delayed onset of obesity and glucose tolerance in interleukin 18 deficient mice by single housed condition. Appl Biol Chem. 2019;62: 1. doi:10.1186/s13765-019-0414-8
16. Liu J, He Z, Ma N, Chen ZY. Beneficial Effects of Dietary Polyphenols on High-Fat Diet-Induced Obesity Linking with Modulation of Gut Microbiota. Journal of Agricultural and Food Chemistry. American Chemical Society; 2020. pp. 33–47. doi:10.1021/acs.jafc.9b06817
17. Panchal SK, Poudyal H, Waanders J, Brown L. Coffee extract attenuates changes in cardiovascular and hepatic structure and function without decreasing obesity in high-carbohydrate, high-fat diet-fed male rats. J Nutr. 2012;142: 690–697. doi:10.3945/jn.111.153577
18. Santos RMM, Lima DRA. Coffee consumption, obesity and type 2 diabetes: a mini-review. European Journal of Nutrition. Dr. Dietrich Steinkopff Verlag GmbH and Co. KG; 2016. pp. 1345–1358. doi:10.1007/s00394-016-1206-0
19. Nishitsuji K, Watanabe S, Xiao J, Nagatomo R, Ogawa H, Tsunematsu T, *et al*. Effect of coffee or coffee components on gut microbiome and short-chain fatty acids in a mouse model of metabolic syndrome. Sci Rep. 2018;8: 16173. doi:10.1038/s41598-018-34571-9
20. Farias-Pereira R, Park CS, Park Y. Mechanisms of action of coffee bioactive components on lipid metabolism. Food Science and Biotechnology. The Korean Society of Food Science and Technology; 2019. pp. 1287–1296. doi:10.1007/s10068-019-00662-0
21. Sousa AG, Da Costa THM. Usual coffee intake in Brazil: Results from the National Dietary Survey 2008-9. Br J Nutr. 2015;113: 1615–1620. doi:10.1017/S0007114515000835
22. Reis CEG, Paiva CLRDS, Amato angélica a., Lofrano-Porto A, Wassell S, Bluck LJC, *et al*. Decaffeinated coffee improves insulin sensitivity in healthy

men. Br J Nutr. 2018;119: 1029–1038. doi:10.1017/S000711451800034X

23. National Research Council of the National Academies. Guide for the Care and Use of Laboratory Animals. 8th ed. The National Academy Press, editor. Washington DC; 2011.

24. Reeves PG, Nielsen FH, George C. Fahey J. AIN-93 Purified Diets for Laboratory Rodents: Final Report of the American Institute of Nutrition Ad Hoc Writing Committee on the Reformulation of the AIN-76A Rodent Diet | The Journal of Nutrition | Oxford Academic. J Nutr. 1993;123: 1939–1951. doi:https://doi.org/10.1093/jn/123.11.1939

25. Vieira VJ, Valentine RJ, Wilund KR, Woods JA. Effects of diet and exercise on metabolic disturbances in high-fat diet-fed mice. Cytokine. 2009;46: 339–345. doi:10.1016/j.cyto.2009.03.006

26. Kanemoto R, Kimura S, Okamura G. Cholesterol lowering effect of soybean lipophilic proteins associated with phospholipids in rat. Soy protein Res. 2007;10: 83–87.

27. Tamura M, Nakagawa H, Hori S, Suzuki T, Hirayama K. Plasma quercetin metabolites are affected by intestinal microbiota of human microbiota-associated mice fed with a quercetin-containing diet. J Clin Biochem Nutr. 2019. doi:10.3164/jcbn.19-45

28. De-Souza MT, Brigido M de M, Maranhão AQ. Técnicas básicas em biologia molecular. 2ª. UnB E, editor. Brasília; 2016.

29. Huijsdens XW, Linskens RK, Mak M, Meuwissen SGM, Vandenbroucke-Grauls CMJE, Savelkoul PHM. Quantification of bacteria adherent to gastrointestinal mucosa by real-time PCR. J Clin Microbiol. 2002;40: 4423–4427. doi:10.1128/JCM.40.12.4423-4427.2002

30. Rinttilä T, Kassinen A, Malinen E, Krogius L, Palva A. Development of an extensive set of 16S rDNA-targeted primers for quantification of pathogenic and indigenous bacteria in faecal samples by real-time PCR. J Appl Microbiol. 2004;97: 1166–1177. doi:10.1111/j.1365-2672.2004.02409.x

31. Nakayama T, Oishi K. Influence of coffee (Coffea arabica) and galacto-oligosaccharide consumption on intestinal microbiota and the host responses. FEMS Microbiol Lett. 2013;343: 161–168. doi:10.1111/1574-6968.12142

32. Haarman M, Knol J. Quantitative real-time PCR analysis of fecal Lactobacillus species in infants receiving a prebiotic infant formula. Appl Environ Microbiol. 2006;72: 2359–2365. doi:10.1128/AEM.72.4.2359-2365.2006

33. Layton A, McKay L, Williams D, Garrett V, Gentry R, Sayler G. Development of Bacteroides 16S rRNA gene taqman-based real-time PCR assays for estimation of total, human, and bovine fecal pollution in water. Appl Environ Microbiol. 2006;72: 4214–4224. doi:10.1128/AEM.01036-05

34. Talarico ST, Santos FE, Brandt KG, Martinez MB, Taddei CR. Anaerobic bacteria in the intestinal microbiota of Brazilian children. Clinics. 2017;72: 154–160. doi:10.6061/clinics/2017(03)05

35. Livak KJ, Schmittgen TD. Analysis of relative gene expression data using real-time quantitative PCR and the 2-ΔΔCT method. Methods. 2001;25: 402–408. doi:10.1006/meth.2001.1262

36. Peinnequin A, Mouret C, Birot O, Alonso A, Mathieu J, Clarençon D, *et al*. Rat pro-inflammatory cytokine and cytokine related mRNA quantification by real-time polymerase chain reaction using SYBR green. BMC Immunol. 2004;5: 3. doi:10.1186/1471-2172-5-3

37. Di Liddo R, Bertalot T, Schuster A, Schrenk S, Tasso A, Zanusso I, *et al*. Anti-

inflammatory activity of Wnt signaling in enteric nervous system: In vitro preliminary evidences in rat primary cultures. J Neuroinflammation. 2015;12: 23. doi:10.1186/s12974-015-0248-1

38. Wang Q, Du F, Qian Z-M, Ge XH, Zhu L, Yung WH, *et al*. Lipopolysaccharide Induces a Significant Increase in Expression of Iron Regulatory Hormone Hepcidin in the Cortex and Substantia Nigra in Rat Brain. Endocrinology. 2008;149: 3920–3925. doi:10.1210/en.2007-1626

39. Cowan TE, Palmnäs MSA, Yang J, Bomhof MR, Ardell KL, Reimer RA, *et al*. Chronic coffee consumption in the diet-induced obese rat: Impact on gut microbiota and serum metabolomics. J Nutr Biochem. 2014;25: 489–495. doi:10.1016/j.jnutbio.2013.12.009

40. Choi BK, Park SB, Lee DR, Lee HJ, Jin YY, Yang SH, *et al*. Green coffee bean extract improves obesity by decreasing body fat in high-fat diet-induced obese mice. Asian Pac J Trop Med. 2016;9: 635–643. doi:10.1016/j.apjtm.2016.05.017

41. Alristina AD, Adi AC, Ismawati R. The Effect of Green Coffee Bean Extract on The Weight Rats with High Fat Diet. Heal Notions. 2018;2: 446–449. Available: http://heanoti.com/index.php/hn/article/view/hn20408/163

42. Rustandi F, Aman IGM, Pinatih GNI. Administration of bali arabica (Coffea arabica) coffee extracts decreases abdominal fat and body weight in obese wistar rats (Rattus norvegicus). Indones J Anti-Aging Med. 2019;3. Available: https://ijaam-unud.org/ojs/index.php/ijaam/article/view/97

43. Vitaglione P, Mazzone G, Lembo V, D'Argenio G, Rossi A, Guido M, *et al*. Coffee prevents fatty liver disease induced by a high-fat diet by modulating pathways of the gut-liver axis. J Nutr Sci. 2019;8. doi:10.1017/jns.2019.10

44. Ilmiawati C, Fitri F, Rofinda ZD, Reza M. Green coffee extract modifies body weight, serum lipids and TNF-α in high-fat diet-induced obese rats. BMC Res Notes. 2020;13. doi:10.1186/s13104-020-05052-y

45. Murase T, Yokoi Y, Misawa K, Ominami H, Suzuki Y, Shibuya Y, *et al*. Coffee polyphenols modulate whole-body substrate oxidation and suppress postprandial hyperglycaemia, hyperinsulinaemia and hyperlipidaemia. Br J Nutr. 2012;107: 1757–1765. doi:10.1017/S0007114511005083

46. An Y, Xu W, Li H, Lei H, Zhang L, Hao F, *et al*. High-fat diet induces dynamic metabolic alterations in multiple biological matrices of rats. J Proteome Res. 2013;12: 3755–3768. doi:10.1021/pr400398b

47. Mercer JG, Archer ZA. Putting the diet back into diet-induced obesity: Diet-induced hypothalamic gene expression. European Journal of Pharmacology. Elsevier; 2008. pp. 31–37. doi:10.1016/j.ejphar.2007.11.077

48. La Fleur SE, Van Rozen AJ, Luijendijk MCM, Groeneweg F, Adan RAH. A free-choice high-fat high-sugar diet induces changes in arcuate neuropeptide expression that support hyperphagia. Int J Obes. 2010;34: 537–546. doi:10.1038/ijo.2009.257

49. de Carvalho Marchesin J, Celiberto LS, Orlando AB, de Medeiros AI, Pinto RA, Zuanon JAS, *et al*. A soy-based probiotic drink modulates the microbiota and reduces body weight gain in diet-induced obese mice. J Funct Foods. 2018;48: 302–313. doi:10.1016/j.jff.2018.07.010

50. Farah A, Duarte G. Bioavailability and Metabolism of Chlorogenic Acids from Coffee. Coffee in Health and Disease Prevention. Elsevier Inc.; 2014. pp. 789–801. doi:10.1016/B978-0-12-409517-5.00087-5

51. Rendón MY, dos Santos Scholz MB, Bragagnolo N. Is cafestol retained on the paper filter in the preparation of filter coffee? Food Res Int. 2017;100: 798–803.

doi:10.1016/j.foodres.2017.08.013

52. da CRUZ RG, Vieira TMF de S, de LIRA SP. Potential antioxidant of brazilian coffee from the region of cerrado. Food Sci Technol. 2018;38: 447–453. doi:10.1590/1678-457x.08017

53. Nora CD, Müller CDR, de Bona GS, Rios A de O, Hertz PF, Jablonski A, *et al*. Effect of processing on the stability of bioactive compounds from red guava (Psidium cattleyanum Sabine) and guabiju (Myrcianthes pungens). J Food Compos Anal. 2014;34: 18–25. doi:10.1016/j.jfca.2014.01.006

54. Wojdyło A, Figiel A, Legua P, Lech K, Carbonell-Barrachina ÁA, Hernández F. Chemical composition, antioxidant capacity, and sensory quality of dried jujube fruits as affected by cultivar and drying method. Food Chem. 2016;207: 170–179. doi:10.1016/j.foodchem.2016.03.099

55. Maki C, Funakoshi-Tago M, Aoyagi R, Ueda F, Kimura M, Kobata K, *et al*. Coffee extract inhibits adipogenesis in 3T3-L1 preadipocyes by interrupting insulin signaling through the downregulation of IRS1. PLoS One. 2017;12: 1–19. doi:10.1371/journal.pone.0173264

56. Feyisa TO, Melka DS, Menon M, Labisso WL, Habte ML. Investigation of the effect of coffee on body weight, serum glucose, uric acid and lipid profile levels in male albino Wistar rats feeding on high-fructose diet. Lab Anim Res. 2019;35: 1–8. doi:10.1186/s42826-019-0024-y

57. Shokouh P, Jeppesen PB, Christiansen CB, Mellbye FB, Hermansen K, Gregersen S. Efficacy of Arabica Versus Robusta Coffee in Improving Weight, Insulin Resistance, and Liver Steatosis in a Rat Model of Type-2 Diabetes. Nutrients. 2019;11: 2074. doi:10.3390/nu11092074

58. Duarte SM da S, Abreu CMP de, Menezes HC de, Paula FB de A, Pereira RGFA, Gouvêa CMCP. Efeito da bebida de café descascado sobre a atividade antioxidante, os parâmetros hematológicos e bioquímicos em ratos. Ciência e Tecnol Aliment. 2009;29: 703–708. doi:10.1590/s0101-20612009000400001

59. Karabudak E, Türközü D, Köksal E. Association between coffee consumption and serum lipid profile. Exp Ther Med. 2015;9: 1841–1846. doi:10.3892/etm.2015.2342

60. Fielding CJ, Fielding PE. Molecular physiology of reverse cholesterol transport. Journal of Lipid Research. Elsevier; 1995. pp. 211–228. doi:10.1016/s0022-2275(20)39898-9

61. Millar CL, Duclos Q, Blesso CN. Effects of dietary flavonoids on reverse cholesterol transport, HDL metabolism, and HDL function. Advances in Nutrition. American Society for Nutrition; 2017. pp. 226–239. doi:10.3945/an.116.014050

62. Burke MF, Khera A V., Rader DJ. Polyphenols and cholesterol efflux: Is coffee the next red wine? Circulation Research. Lippincott Williams & Wilkins; 2010. pp. 627–629. doi:10.1161/CIRCRESAHA.109.215855

63. Luo J, Yang H, Song BL. Mechanisms and regulation of cholesterol homeostasis. Nature Reviews Molecular Cell Biology. Nature Research; 2020. pp. 225–245. doi:10.1038/s41580-019-0190-7

64. Yokota A, Fukiya S, Islam KBMS, Ooka T, Ogura Y, Hayashi T, *et al*. Is bile acid a determinant of the gut microbiota on a high-fat diet? Gut Microbes. 2012;3: 455–459. doi:10.4161/gmic.21216

65. Ajouz H, Mukherji D, Shamseddine A. Secondary bile acids: An underrecognized cause of colon cancer. World Journal of Surgical Oncology. BioMed Central Ltd.; 2014. doi:10.1186/1477-7819-12-164

66. Ocvirk S, O'Keefe SJ. Influence of Bile Acids on Colorectal Cancer Risk: Potential Mechanisms Mediated by Diet-Gut Microbiota Interactions. Current Nutrition Reports. Current Science Inc.; 2017. pp. 315–322. doi:10.1007/s13668-017-0219-5
67. Ontawong A, Duangjai A, Muanprasat C, Pasachan T, Pongchaidecha A, Amornlerdpison D, *et al*. Lipid-lowering effects of Coffea arabica pulp aqueous extract in Caco-2 cells and hypercholesterolemic rats. Phytomedicine. 2019;52: 187–197. doi:10.1016/j.phymed.2018.06.021
68. Post SM, De Wit ECM, Princen HMG. Cafestol, the cholesterol-raising factor in boiled coffee, suppresses bile acid synthesis by downregulation of cholesterol 7α-hydroxylase and sterol 27-hydroxylase in rat hepatocytes. Arterioscler Thromb Vasc Biol. 1997;17: 3064–3070. doi:10.1161/01.ATV.17.11.3064
69. Li Y, Rahman SU, Huang Y, Zhang Y, Ming P, Zhu L, *et al*. Green tea polyphenols decrease weight gain, ameliorate alteration of gut microbiota, and mitigate intestinal inflammation in canines with high-fat-diet-induced obesity. J Nutr Biochem. 2020;78: 108324. doi:10.1016/j.jnutbio.2019.108324
70. Jin J-S, Touyama M, Hisada T, Benno Y. Effects of green tea consumption on human fecal microbiota with special reference to *Bifidobacterium* species. Microbiol Immunol. 2012;56: 729–739. doi:10.1111/j.1348-0421.2012.00502.x
71. Kim YA, Keogh JB, Clifton PM. Polyphenols and Glycemic Control. Nutrients. 2016;8: 17. doi:10.3390/nu8010017
72. Rossi E, Cimdins A, Lüthje P, Brauner A, Sjöling Å, Landini P, *et al*. "It's a gut feeling"–Escherichia coli biofilm formation in the gastrointestinal tract environment. Critical Reviews in Microbiology. Taylor and Francis Ltd; 2018. pp. 1–30. doi:10.1080/1040841X.2017.1303660
73. Eckburg PB, Bik EM, Bernstein CN, Purdom E, Dethlefsen L, Sargent M, *et al*. Microbiology: Diversity of the human intestinal microbial flora. Science (80-). 2005;308: 1635–1638. doi:10.1126/science.1110591
74. Million M, Thuny F, Angelakis E, Casalta JP, Giorgi R, Habib G, *et al*. Lactobacillus reuteri and Escherichia coli in the human gut microbiota may predict weight gain associated with vancomycin treatment. Nutr Diabetes. 2013;3: e87–e87. doi:10.1038/nutd.2013.28
75. Dadi PK, Ahmad M, Ahmad Z. Inhibition of ATPase activity of Escherichia coli ATP synthase by polyphenols. Int J Biol Macromol. 2009;45: 72–79. doi:10.1016/j.ijbiomac.2009.04.004
76. Pagliarulo C, De Vito V, Picariello G, Colicchio R, Pastore G, Salvatore P, *et al*. Inhibitory effect of pomegranate (Punica granatum L.) polyphenol extracts on the bacterial growth and survival of clinical isolates of pathogenic Staphylococcus aureus and Escherichia coli. Food Chem. 2016;190: 824–831. doi:10.1016/j.foodchem.2015.06.028
77. Karar MGE, Pletzer D, Jaiswal R, Weingart H, Kuhnert N. Identification, characterization, isolation and activity against Escherichia coli of quince (Cydonia oblonga) fruit polyphenols. Food Res Int. 2014;65: 121–129. doi:10.1016/j.foodres.2013.10.040
78. Matsumoto M, Kibe R, Ooga T, Aiba Y, Kurihara S, Sawaki E, *et al*. Impact of intestinal microbiota on intestinal luminal metabolome. Sci Rep. 2012;2: 1–10. doi:10.1038/srep00233
79. Jandhyala SM, Talukdar R, Subramanyam C, Vuyyuru H, Sasikala M, Reddy DN. Role of the normal gut microbiota. World J Gastroenterol. 2015;21: 8836–8847. doi:10.3748/wjg.v21.i29.8787

80. Price CE, Reid SJ, Driessen AJM, Abratt VR. The Bifidobacterium longum NCIMB 702259T ctr gene codes for a novel cholate transporter. Appl Environ Microbiol. 2006;72: 923–926. doi:10.1128/AEM.72.1.923-926.2006
81. Ruiz L, Zomer A, O'Connell-Motherway M, van Sinderen D, Margolles A. Discovering novel bile protection systems in Bifidobacterium breve UCC2003 through functional genomics. Appl Environ Microbiol. 2012;78: 1123–1131. doi:10.1128/AEM.06060-11
82. Ruiz L, O'Connell-Motherway M, Zomer A, de los Reyes-Gavilán CG, Margolles A, van Sinderen D. A bile-inducible membrane protein mediates bifidobacterial bile resistance. Microb Biotechnol. 2012;5: 523–535. doi:10.1111/j.1751-7915.2011.00329.x
83. Fernández-Real JM, Ricart W. Insulin resistance and chronic cardiovascular inflammatory syndrome. Endocrine Reviews. Endocrine Society; 2003. pp. 278–301. doi:10.1210/er.2002-0010
84. Hang D, Kværner AS, Ma W, Hu Y, Tabung FK, Nan H, *et al*. Coffee consumption and plasma biomarkers of metabolic and inflammatory pathways in US health professionals. Am J Clin Nutr. 2019;109: 635–647. doi:10.1093/ajcn/nqy295
85. Kittana H, Gomes-Neto JC, Heck K, Geis AL, Segura Muñoz RR, Cody LA, *et al*. Commensal Escherichia coli Strains can promote intestinal inflammation via differential interleukin-6 production. Front Immunol. 2018;9. doi:10.3389/fimmu.2018.02318
86. Wang S, Moustaid-Moussa N, Chen L, Mo H, Shastri A, Su R, *et al*. Novel insights of dietary polyphenols and obesity. Journal of Nutritional Biochemistry. J Nutr Biochem; 2014. pp. 1–18. doi:10.1016/j.jnutbio.2013.09.001
87. Juge-Aubry CE, Henrichot E, Meier CA. Adipose tissue: A regulator of inflammation. Best Practice and Research: Clinical Endocrinology and Metabolism. Baillière Tindall; 2005. pp. 547–566. doi:10.1016/j.beem.2005.07.009
88. Caro-Gómez E, Sierra J, Escobar J, Álvarez-Quintero R, Naranjo M, Medina S, *et al*. Green Coffee Extract Improves Cardiometabolic Parameters and Modulates Gut Microbiota in High-Fat-Diet-Fed ApoE-/- Mice. Nutrients. 2019;11: 497. doi:10.3390/nu11030497

Supporting information

S1 Table. *P*-values of the statical analysis of microbial composition and bile acid concentration in feces of rats fed control diet without FCS CT (-), control diet + FCS CT (+), high fat diet HF (-) or high fat diet + FCS (HF +). D=diet; FCS= freeze-dried coffee solution; T= time; SEM= Standard error of the mean.

Click here to access/download;Figure;Fig1.tif

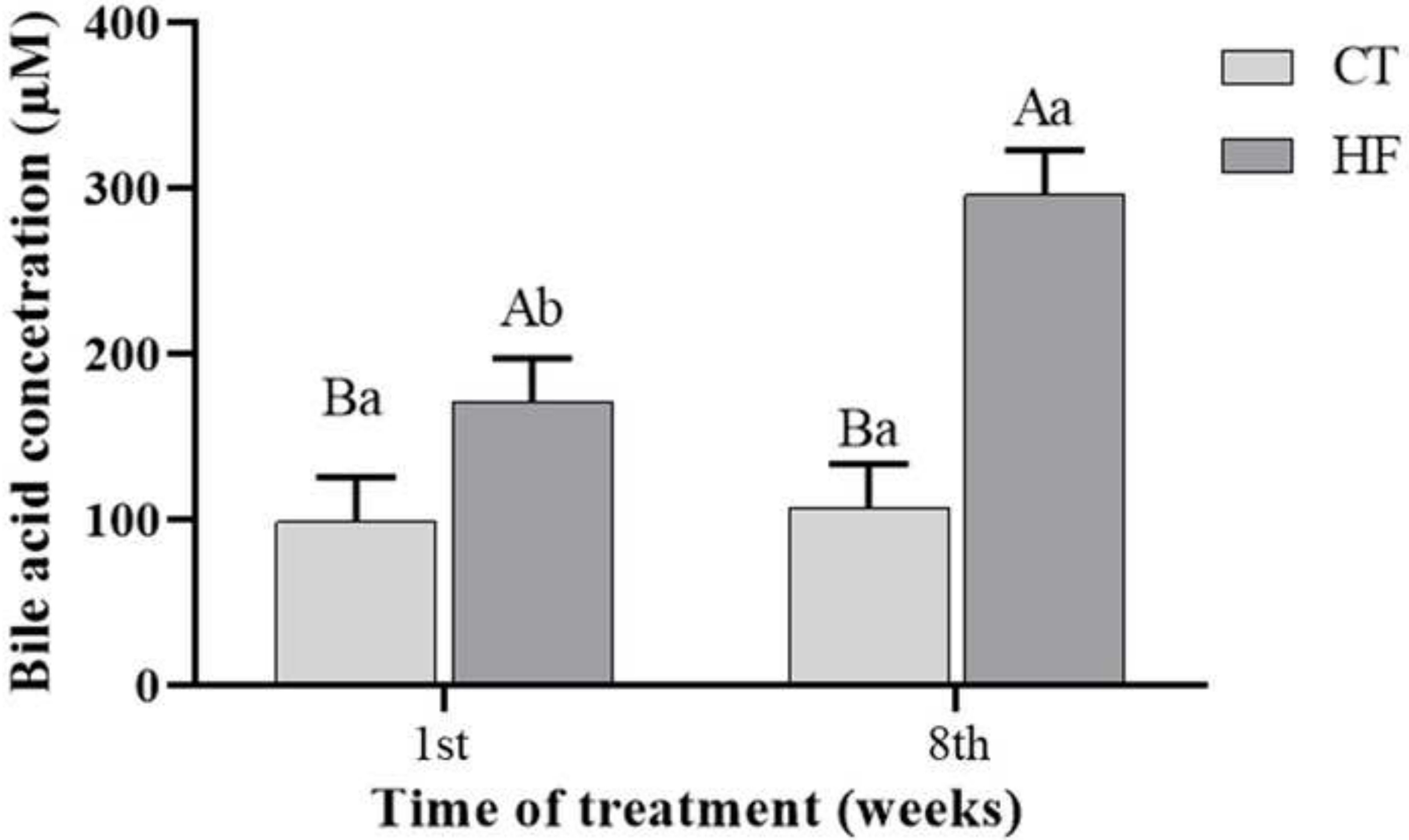

Fig2

Click here to access/download;Figure;Fig2.tif

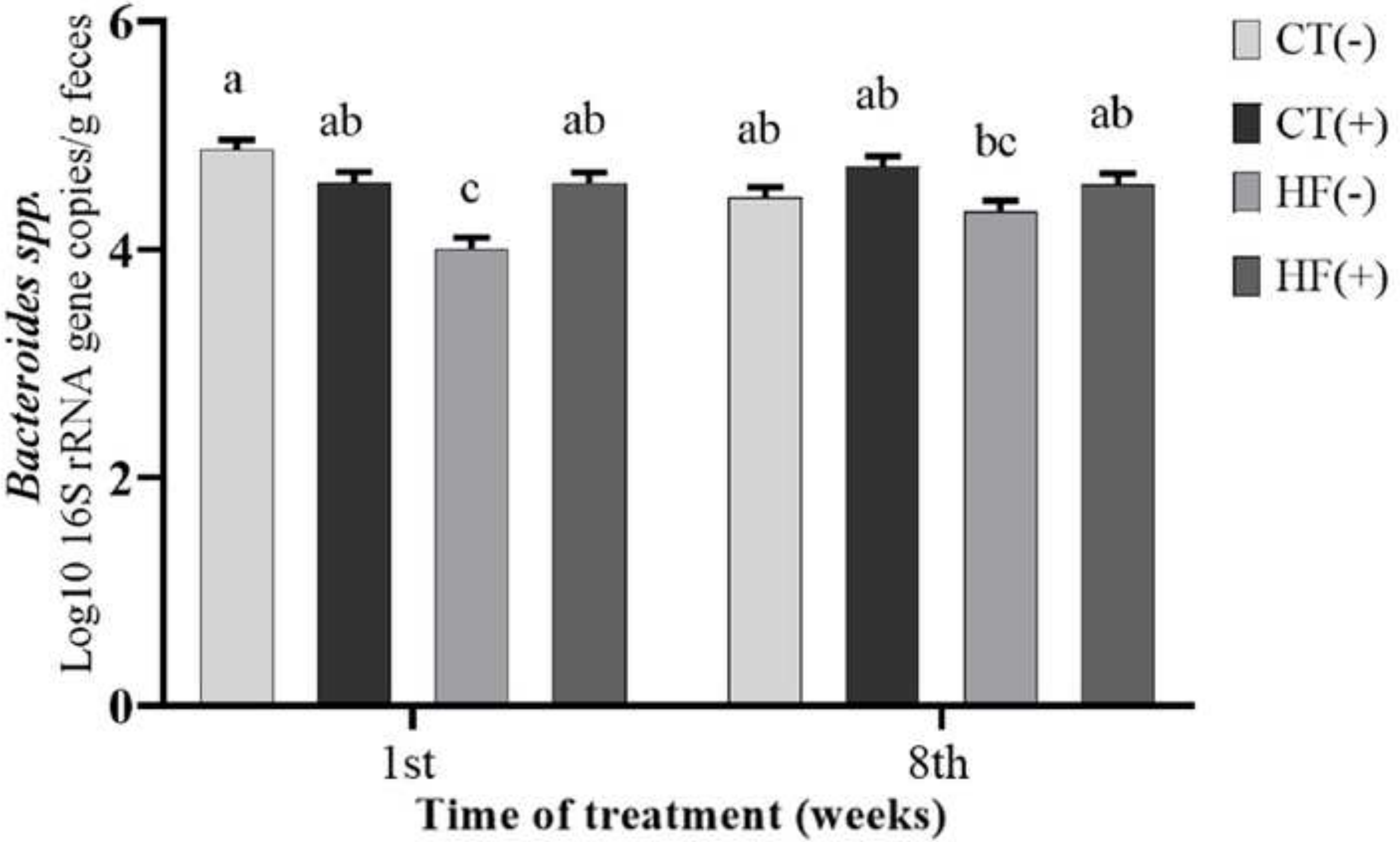

Fig3

Click here to access/download;Figure;Fig3.tif

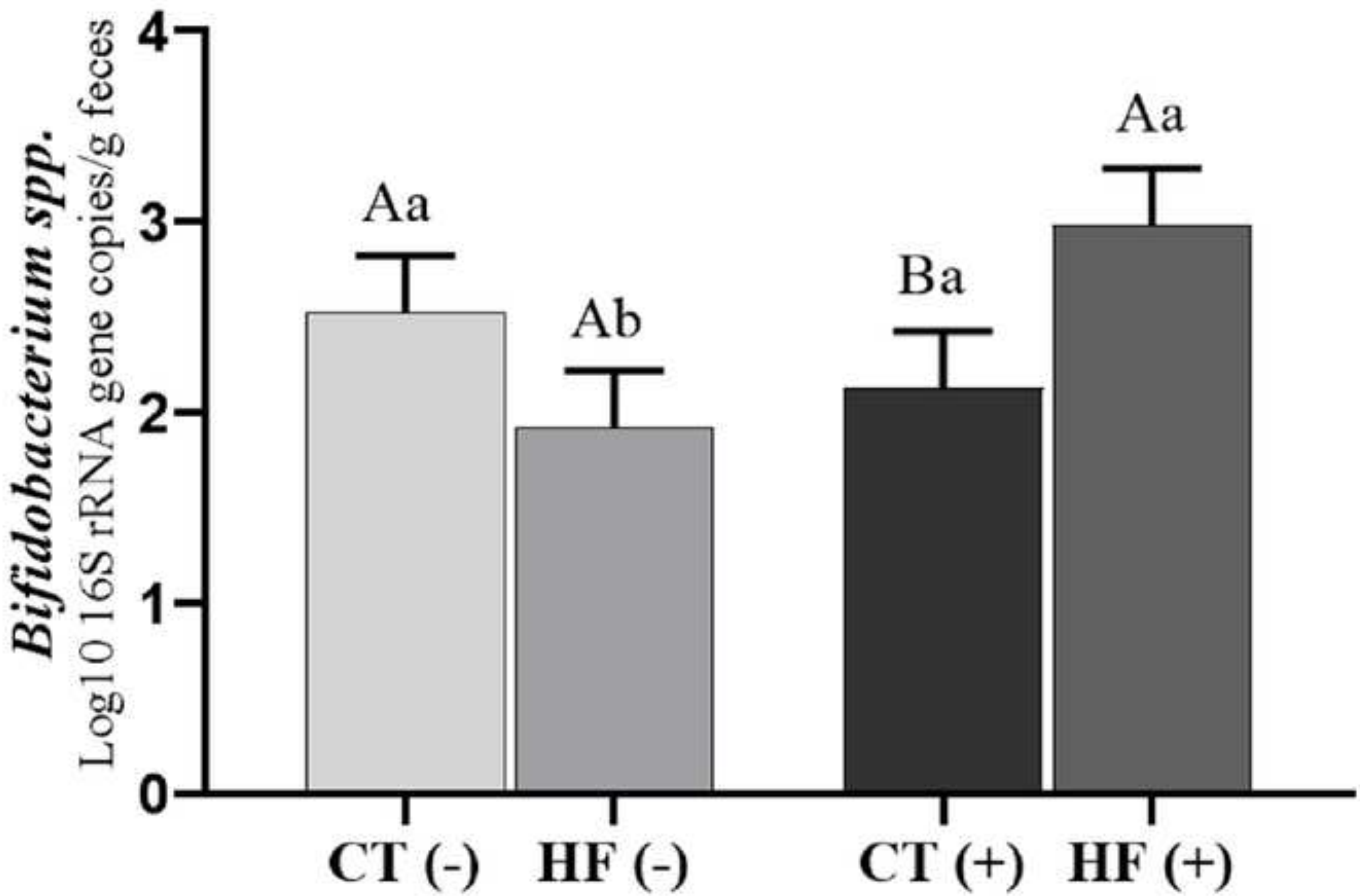

Fig4

Click here to access/download;Figure;Fig4.tif

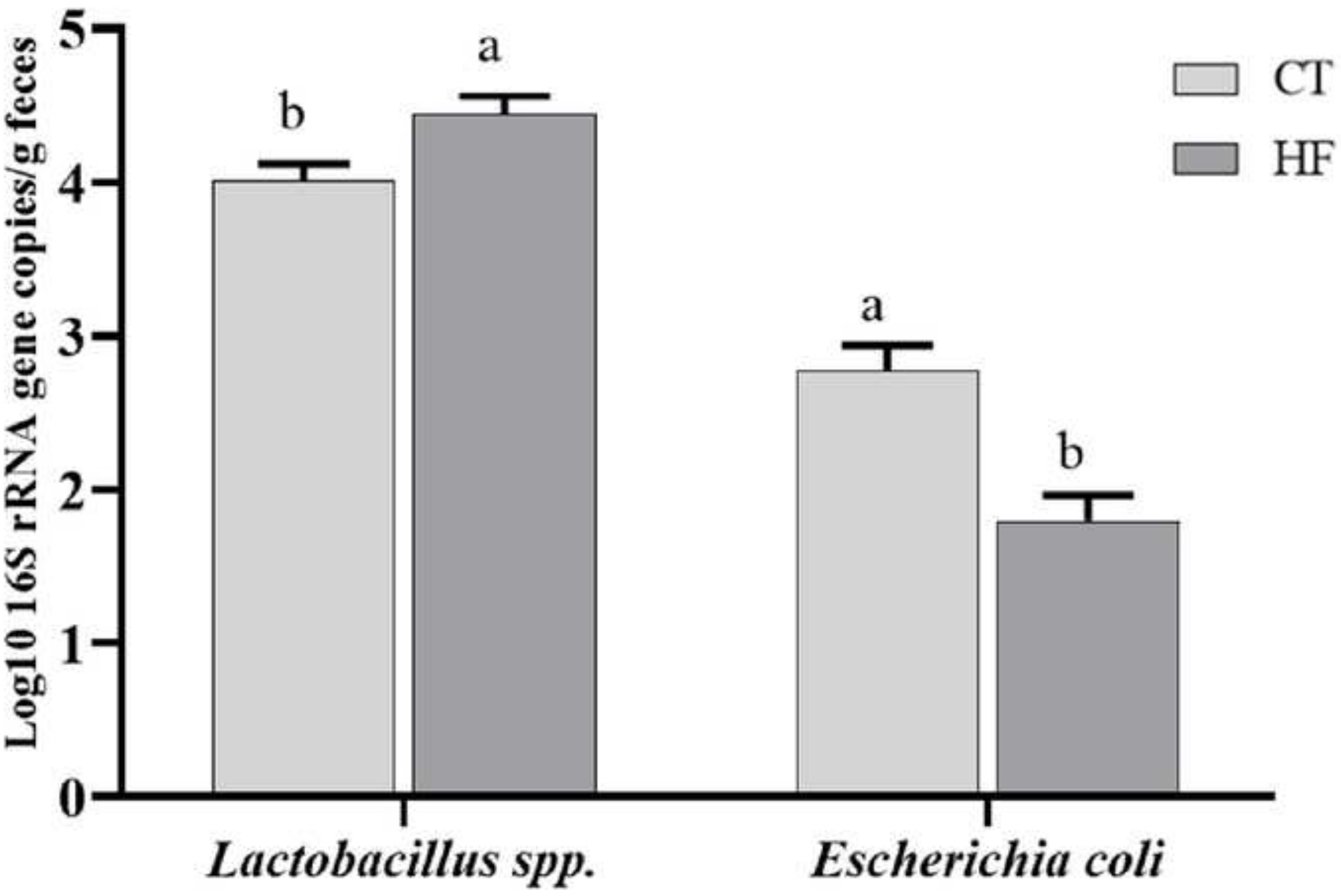

Fig5

Click here to access/download;Figure;Fig5.tif

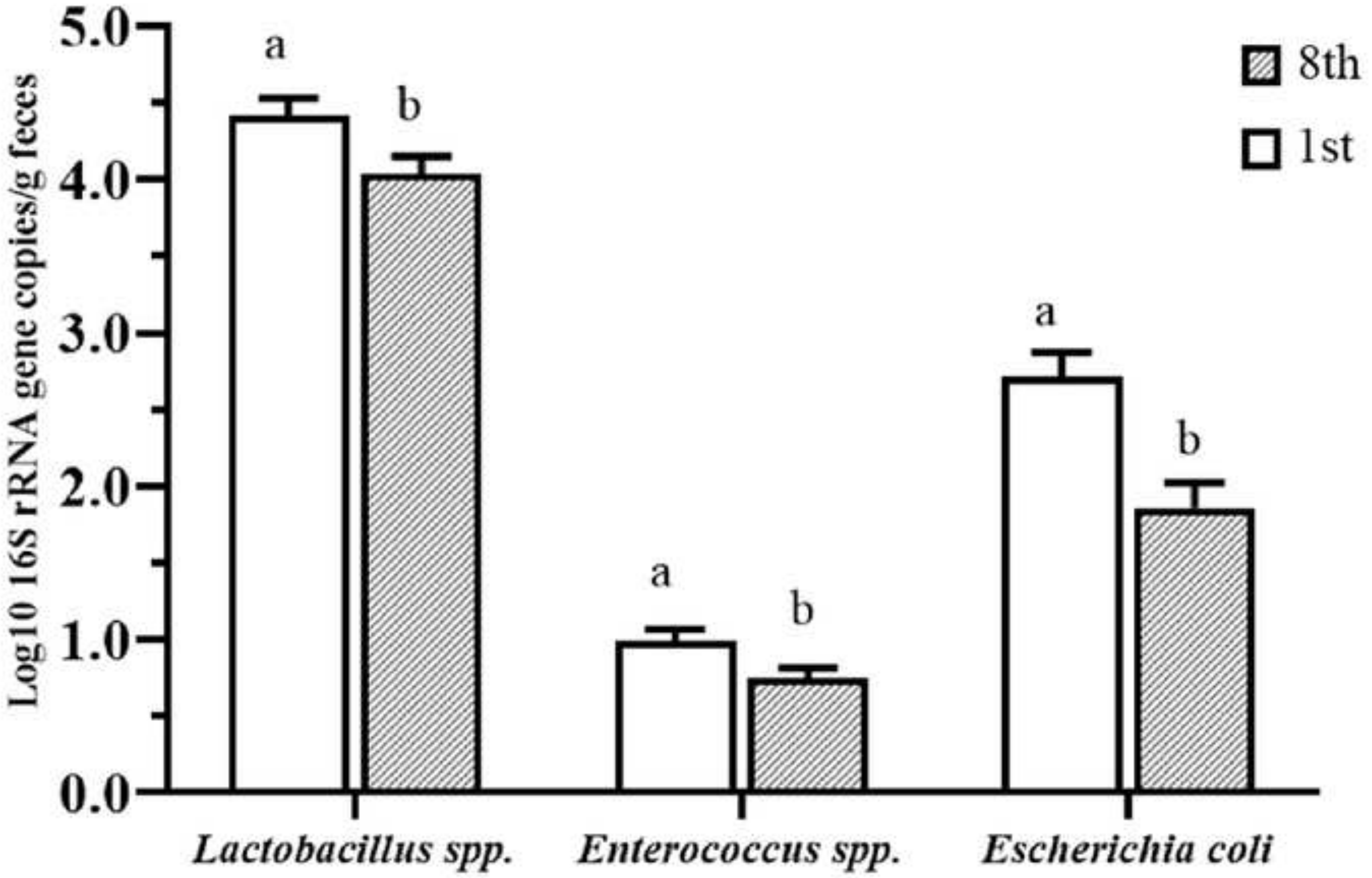

Supplement: Attachment — Submitted filename: PONE-D-21-14462.pdf [file pone.0262270.s003.pdf]
