# Supplementary material for: Effect of a freeze-dried coffee solution in a high-fat diet-induced obesity model in rats: Impact on inflammatory response, lipid profile, and gut microbiota
Source: PLoS One. 2022 Jan 26;17(1):e0262270. doi: 10.1371/journal.pone.0262270 (PMC8791513; doi:10.1371/journal.pone.0262270)
Supplement: Attachment — Submitted filename: Letter Editor.docx [file pone.0262270.s005.docx]

Brasília, December 16, 2021

**To:**	Baochuan Lin, Ph.D.

Academic Editor

PLOS ONE

**Subject:** Revised version of the manuscript PONE-D-21-14462R1

Dear Ph.D. Baochuan Lin,

Thank you by the opportunity to submit a revised version of our manuscript “Effect of freeze-dried coffee solution in a high-fat diet-induced obesity model in rats: biochemical and inflammatory impacts and effects on gut microbiota”. Below are the responses to each specific comment brought up. All changes made were marked on the revised manuscript copy, and the answers to editor questions are presented below. According to editor suggestion, the manuscript was copy-edited for language usage, spelling, and grammar by Editage (editage.com.br). Below is the Editing Certificate issued by Editage.

Yours sincerely,

Marilia Cavalcanti, M.Sc.

Corresponding author

Postgraduate Program in Human Nutrition, Faculty of Health Sciences, Campus Universitário Darcy Ribeiro, Universidade de Brasília, Brasília, POBox 70910-900, Brazil. Phone +(55) - 61 - 31070092 e-mail: mariliaunb@outlook.com

Editor specific comments:

Specific comments:

1. Line 38, replace "Independently" with "Regardless". Accepted.

2. Line 126, change "*Coffea arabica*" to "*C. arabica*". Accepted.

3. Line 127 - 128, there are 4 numbers here with only 3 caffeoylquinic acid listed. Please clarify. It was revised.

4. Line 132, replace "n°" with "no." It was revised.

5. Line 176 - 177, change "San Luis" to "St. Louis". Accepted.

6. Line 199, This is confusing.  Is 10μl the total reaction volume? Please rephrase for clarity. It was revised.

7. Line 211 - 214, please follow scientific nomenclature rules and use abbreviation here since these bacteria were mentioned earlier. For example:  *E. coli, E. faecalis, B. lactis*, & *L. plantarum.* It was revised.

8. Line 407, change "independently" to "independent" It was revised.

9. Line 445, change “*Coffeea*” to “*C.*” Accepted.

10. Line 530, change "pathogen bacteria" to pathogenic bacteria" It was revised.

11. Line 558 - 559, replace “*Bifidobacterium*” to “*B.*” Accepted.

12. Line 597 - 599, the statement "..., while some health..." was not supported by the data presented, please delete. It was deleted.
